# Clonal Expansion in Multiple *Phyllosticta* Species Causing Citrus Black Spot or Similar Symptoms in China

**DOI:** 10.3390/jof9040449

**Published:** 2023-04-06

**Authors:** Wen Wang, Tao Xiong, Yating Zeng, Wenwen Li, Chen Jiao, Jianping Xu, Hongye Li

**Affiliations:** 1 The Key Laboratory of Molecular Biology of Crop Pathogens and Insects of Ministry of Agricultural, The Key Laboratory of Biology of Crop Pathogens and Insects of Zhejiang Province, Institute of Biotechnology, Zhejiang University, 866 Yuhangtang Road, Hangzhou 310058, China; 2 Department of Biochemistry, Genetics and Microbiology, Forestry and Agricultural Biotechnology Institute (FABI), University of Pretoria, Pretoria 0028, South Africa; 3 Department of Biology, McMaster University, Hamilton, ON L8S 4K1, Canada

**Keywords:** citrus disease, clonal dispersal, diversity, multi-gene phylogeny, pathogenicity, *Phyllostictaceae*

## Abstract

*Phyllosticta* spp. are important pathogens of citrus plants. Several *Phyllosticta* species associated with *Citrus* species grown in China have been reported; however, the relative prevalences of individual species and the distributions of their genotypes among host *Citrus* species remain largely unknown. In this study, we conducted an extensive survey of *Phyllosticta* species across 11 citrus-producing provinces in southern China. From fruits and leaves with black spots or black-spot-like symptoms, a total of 461 *Phyllosticta* strains were isolated. Based on molecular (ITS, *actA*, *tef1*, *gapdh*, LSU, and *rpb2* sequences) and morphological data, the strains were systematically identified as belonging to five species: *P. capitalensis*, *P. citrichinaensis*, *P. citriasiana*, *P. citricarpa*, and *P. paracitricarpa*. To further understand intraspecific genetic diversity and relationships, strains of five species from different geographic and host sources were analyzed based on the multilocus sequence data. Our population genetic analyses revealed that all five *Phyllosticta* species on citrus showed evidence for clonal dispersals within and among geographic regions. In addition, pathogenicity tests using representative strains showed that all five species can cause disease on the tested *Citrus* spp. We discuss the implications of our results for the control and management of Citrus Black Spot and related diseases.

## 1. Introduction

*Phyllosticta* is a widely distributed genus of plant pathogens that can infect a diverse range of host plants, including citrus, banana, and grape [1,2,3,4,5,6,7]. Citrus are important fruits grown in more than 140 countries [8], but its production in some areas has been severely impacted by fungal pathogens such as *P. citricarpa*, which causes Citrus Black Spot (CBS). CBS is a foliar and fruit disease that affects various *Citrus* spp. [9,10,11,12]. This pathogen is present in most citrus-producing areas worldwide and can cause several types of spot symptoms. In the European Union, CBS is classified as an A1 quarantine pest and significantly impacts the global trade of fresh citrus fruits [7].

Apart from *P. citricarpa*, seven other species of *Phyllosticta* have been linked to citrus, such as *P. capitalensis*, which is commonly reported as an endophyte or weak pathogen with a broad host range and distribution across different regions [13,14,15] *P. citriasiana* causes Citrus Tan Spot disease specifically in *C. maxima* in Asia [16], while *P. citribraziliensis* is associated with *Citrus* species in Brazil [14]. *P. citrichinaensis* causes freckle spot in China [17], *P. citrimaxima* causes Citrus Tan Spot on the fruit of *C. maxima* in Thailand [18], *P. paracapitalensis* is found in Italy, Spain, and New Zealand [2], and *P. paracitricarpa* is present in China and Greece [2,17]. *Phyllosticta* species have been linked to various citrus diseases worldwide and their geographic distributions and host ranges vary among different species.

In China, where citrus is an important agricultural crop, five of eight *Phyllosticta* species associated with citrus have been reported from various *Citrus* varieties [17,19]. The five species are *P. capitalensis*, *P. citriasiana*, *P. citricarpa*, *P. citrichinaensis*, and *P. paracitricarpa*, with *P. paraciticarpa* being recently elevated from a subclade of *P. citricarpa* to a separate species based on sequence divergences at six loci [2,17].

Understanding the distribution and genetic relationships of *Phyllosticta* species on citrus in China is critical for managing these pathogens. However, while there has been an increase in using molecular markers for identifying *Phyllosticta* species, the relative abundance of individual species in this genus across large geographic regions in China remains largely unknown [2,17,18]. Moreover, previous studies have focused mainly on species diversity, little attention has been paid to the genetic relationships between individuals within species [14,16,17,18,19]. Therefore, continued molecular taxonomy and genetic research are necessary to accurately understand the relationships between and within different *Phyllostitca* species on citrus in China.

Given that China is one of the origin countries of citrus, the co-evolutionary history between citrus and *Phyllosticta* species could have contributed to speciation and specialization [20,21,22]. On the other hand, the recent rapid expansion of citrus cultivation across many parts of China could also lead to shared species and strains of *Phyllosticta* among geographically distant citrus plantations. Despite the importance of understanding and managing the distribution and pathogenicity of *Phyllosticta* in citrus, relatively little is known about the genetic relationships between individuals within species [17,19,23,24].

To fill this knowledge gap, we broadly surveyed citrus leaves and fruits for symptoms of black spots or similar symptoms in southern China. We obtained and purified all *Phyllosticta*-like colonies to investigate the distributions of species and genotypes of *Phyllosticta* in citrus plantations. Species affiliations of all strains were analyzed using the latest six housekeeping barcoding for *Phyllosticta* taxa as recommended by Guarnaccia et al. [2]. For representative subsets of the isolates, their multilocus genotypes were determined based on SNPs within these six loci. The pathogenicity of five species to citrus was also evaluated. This study provides a comprehensive understanding of *Phyllosticta* distributions and potential threats on citrus in China and offers insights into citrus management strategies to reduce the impact of these pathogens.

## 2. Materials and Methods

### 2.1. Samples and Fungal Isolates

Disease surveys in citrus-producing regions were conducted from 2009 to 2021. Citrus fruits and leaves with black spot or similar symptoms were collected from cultivated *Citrus* species such as mandarins, oranges, pomelos, and lemons in major citrus-producing regions, including Chongqing Municipality and Fujian, Guangdong, Guangxi, Guizhou, Hubei, Hunan, Jiangxi, Sichuan, Yunnan, and Zhejiang provinces. The collected samples were treated as previously described [2,17]. A small piece of tissue (5 × 5 mm^2^) was aseptically cut from the margin of infected fruits or leaves, surface sterilized by 1% sodium hypochlorite solution for 45 s, followed by 70% ethanol solution for 30 s, and by sterilized water three times, and finally dried in sterilized tissue paper. The sections were incubated at 25 °C on 1/2 potato dextrose agar (PDA), supplemented with 100 µg/mL penicillin and 100 µg/mL ampicillin until colonies developed (about 9–12 days). To obtain pure cultures, single hyphal tips from the colonies were transferred to 2% PDA. Data for selected isolates are listed in Supplementary Table S1.

### 2.2. DNA Extraction, PCR Amplification, and Sequencing

Genomic DNA was extracted using the CTAB protocol described by van Burik et al. [25]. Before DNA extraction, selected isolates were grown on PDA at 25 ± 2 °C for 14 d. The activated mycelia of each isolate were scraped from the surface of the PDA medium with a sterile scalpel and transferred into 2 mL centrifuge tubes. The extracted DNA was suspended in 30 µL sterilized ultrapure water for an hour and evaluated using a Nano-100 micro-spectrophotometer (Hangzhou Allsheng Instruments, Hangzhou, China).

Six pairs of primers were used to amplify partial regions of six loci. Primers V9G [25] and ITS4 [26] were used to amplify the internal transcribed spacer region of the nuclear ribosomal RNA operon (ITS), including the 3’ end of the 18S rRNA, the first ITS region, the 5.8S rRNA gene, the second ITS region, and the 5’ end of the 28S rRNA gene. To amplify a partial fragment of the actin encoding gene (*actA*), primers ACT-512F and ACT-783R [27] were used. To amplify partial translation elongation factor 1-α gene (*tef1*), primers EF1-728F [27] and EF2 [28] were used. Glyceraldehyde-3-phosphate dehydrogenase (gapdh) was amplified using primers Gpd1-LM and Gpd2-LM [29]. For *P. citricarpa* and *P. citriasiana* isolates, the alternative primers Gpd1 [30] and GPDHR2 [14] were used to amplify *gapdh* [31]. To amplify the 28S large subunit rDNA (LSU), primers LROR [32] and LR5 were used. The RNA polymerase II second largest subunit (*rpb2*) was amplified with RPB2-5F2 [32] and fRPB2-7cR [33]. The PCR amplification mixtures and cycling conditions for ITS, *actA*, *tef1*, *gapdh*, LSU, and *rpb2* followed Glienke et al. [14]. All PCR products were sequenced in both directions, and ITS sequencing reactions were performed by Zhejiang Sunya Biotechnology Co. LTD. Sequencing reactions for the other five loci were performed by Beijing Genomics Institute of Guangzhou, China. The nucleotide sequences were read and edited using Geneious version 7.1.8 [34]. Sequences have been submitted to GenBank repository (http://www.ncbi.nlm.nih.gov; accessed on 11 December 2022) and the GenBank accession numbers are shown in Supplementary Table S1. 

### 2.3. Phylogenetic Analyses

Sequences generated in this study were compared and analyzed with strains of closely related *Phyllosticta* species and downloaded from GenBank. The sequences for a total of 65 isolates were downloaded (Supplementary Table S1) and analyzed with our own sequences. All sequences, including ITS, *actA*, *tef1*, *gapdh*, LSU, and *rpb2* regions, as well as the combination of the six sequence regions, were aligned using MAFFT program [35]. with the FFT-NS-I algorithm, and edited manually using MEGA v. 6.0.5 software [36].

Phylogenetic analyses of single loci and a concatenated matrix of the six loci were performed using maximum likelihood (ML) and maximum parsimony (MP) methods. PhyML v. 3.1 was used for the ML analyses for each dataset [37]. The software package jModeltest v. 1.2.5 was used to determine the best nucleotide substitution model for each dataset [38]. In PhyML, the maximum number of retained trees was set to 1000, and branch support was determined by non-parametric bootstrapping with 1000 replicates. PAUP v. 4.0 b10 [39] was used for partition homogeneity test (PHT) and MP analyses, with gaps treated as the fifth character. Uninformative characters were excluded, and informative characters were unordered and of equal weight with 1000 random addition replicates. The most parsimonious trees were obtained using the heuristic search function with stepwise addition and tree bisection and reconnection branch swapping. Maxtrees were set to 5000, and zero-length branches were collapsed. A bootstrap analysis (50% majority rule, 1000 replicates) was done to determine statistical support for the internal nodes in the trees. Tree length (TL), consistency index (CI), retention index (RI), and homoplasy index (HI) were used to assess the trees [40]. Phylogenetic trees were viewed using MEGA v. 6.0.5 software [36]. Sequence alignments and phylogenetic trees were deposited in TreeBASE (www.treebase.org; accessed on 20 October 2022).

### 2.4. Morphology

The *Phyllosticta* fungi collected in this study were compared with previously published *Phyllosticta* spp. on citrus [2,14,16,17,18]. To study the morphological characteristics of our isolates and to identify potential novel species in our collection, pine needle agar (PNA) [41] was used to induce sporulation under light at 27 °C. After 30-d incubation, the induced sporocarps were removed from the pine needles under a SMZ800 dissecting microscope (Nikon, Tokyo, Japan) and then embedded in Leica Bio-systems Tissue Freezing Medium (Leica Biosystems Nussloch GmbH, Nussloch, Germany) and sectioned (8 µm thick) using a Cryostar nx50 (Microm International GmbH, Thermo Fisher Scientific, Walldorf, Germany) at −20 °C to observe stromata and stromatic tissue. Conidiophores (conidiogenous cells), spermatogenous cells, conidia, and spermatia were measured after crushing the sporocarps on microscope slides in sterilized water.

Morphological characteristics of our strains in this study were compared with published *Phyllosticta* species. Measurements were recorded using an Eclipse 80i microscope (Nikon, Japan) and a DS-Fi1 digital camera with NIS-Elements F 4.0 software (Nikon, Japan). Records were measured using the latest version of ImageJ software (National Institutes of Health, USA). The results are presented as (minimum–) (mean − standard deviation) − (mean + standard deviation) (–maximum).

Colony color and growth rate were investigated on malt extract agar (MEA), oatmeal agar (OA), and PDA medium as described by Crous et al. [41]. Colony color was determined using the charts of Rayner [42]. Colony growth rates were assessed at 9–39 °C at 3 °C intervals, three plates were used for each media, and two measurements of colony diameter perpendicular to each culture were made after 3, 6, 9, and 12 d of incubation in the dark, after which averages were computed.

### 2.5. Population Genetic Analyses

To investigate if geographic or host populations of individual *Phyllosticta* species were genetically subdivided, strains genotyped according to six loci sequences, were separated into different geographic and host tree-based populations and analyzed using the GenAlEx V.6.5 program [43]. To prevent errors resulting from chance events, subgroups containing only one individual were excluded before analysis. For each of the species, two types of datasets were related: non-clone-corrected (NCC) and clone-corrected (CC) [44]. The NCC datasets included all isolates with genotype information. For the CC datasets, only one representative strain of each multilocus sequence type from each geographic region was included in our analyses. Similarly, for species with isolates from multiple host trees, the potential contributions of host tree species to the total genetic variations were also estimated using GenAlEx for CC datasets. For each species of the dataset, we separately conducted the analyses of molecular variance (AMOVA). Statistical significance for each test was obtained by comparing the observed with the distributions of 999 permutated datasets generated based on a null hypothesis of no genetic differentiations within each analyzed dataset.

In addition, the MLST dataset of each species was also used to identify potential evidence for recombination within individual species. For this test, both the NCC and CC datasets were analyzed. Specifically, linkage disequilibrium analyses were performed in package poppr with 999 permutations [44], and proportion of phylogenetic compatibility pairs of loci was calculated using the program MultiLocus V1.3 with 1000 randomizations for each dataset [45].

### 2.6. Pathogenicity

One isolate of each species identified in this study was inoculated into mature fruit of *C. maxima* cv. Guanximiyou or *C. limon* following the method described by Perryman et al. [46]. Fruits were washed and surface-disinfected by immersion in 70% ethanol for 10 min, and rinsed twice with sterile water. A suspension of conidia (1.0 × 10^5^ conidia/mL) was obtained from cultures grown on PDA at 27 °C. Conidial suspension of each isolate (60 µL) was injected into 6–15 inoculation points on the surface (about 2 mm in deep) of test fruits. The same volume of sterile water was inoculated as the control. Each isolate was inoculated into three fruits as biological replicates. The inoculated fruits were incubated at 25 °C, with a 12 h photoperiod, and 100% relative humidity for lesion development. Lesions area were measured with the latest version of ImageJ software. Re-isolation of inoculated fungi was conducted, and the isolated strain was confirmed by sequencing ITS. The experiment was repeated twice.

## 3. Results

### 3.1. Disease Symptoms and Fungal Isolates

**Figure 1:**
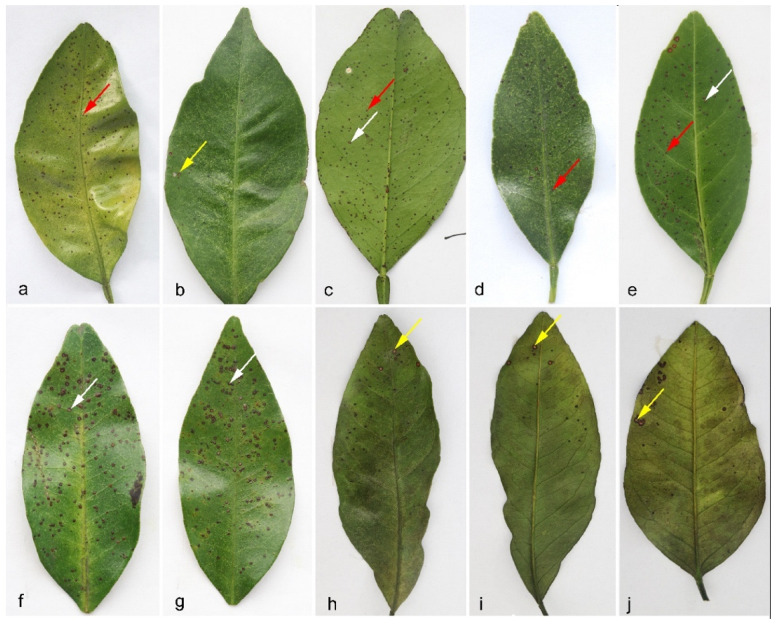
Symptoms of citrus leaf spots from which the *Phyllostitca* isolates were obtained. (**a**–**c**) *C. sinensis*, (**d**–**g**) *C. reticulata*, (**h**–**j**) *C. unishiu*. Red arrow, spot raised and hard, similar but bigger than melanose; white arrow, flat spot, with dark brown margin, gray-white and slightly sunken center; yellow arrow, spots with margin significantly raised, dark brown, and with gray-white, sunken center, similar to “hard spot” of black spot.

Citrus fruits and leaves showing black spot or black spot-like symptoms (Figure 1 and Figure S1) were collected from the main citrus-producing regions in China during the years of 2009 to 2021. The lesions observed on leaves were usually small and round, with diameters not exceeding 3 mm. In general, the spots could be divided into three types. Type I symptoms are similar to those reported for *P. citrichinaensis* [17]. These spots were red-brown, raised, and hard, and similar but usually bigger than the spots of melanose (Figure 1, red arrows). Type II symptoms have spots that were usually flat, with a dark brown margin and a gray-white and slightly sunken center (Figure 1, white arrows). Type III symptoms have spots with significantly raised margins, that were dark brown and with a gray-white, sunken center, similar to “hard spot” of black spot disease (Figure 1, yellow arrows). However, no black dots (pycnidia) were observed in these spots. The lesions observed on fruits showed the typical symptoms of black or tan spot disease (Figure S1). A total of 461 single hyphae isolates showing signatures of *Phyllosticta* were obtained. Detailed information for the isolates, host trees, and geographic locations are listed in Supplementary Table S1.

### 3.2. Phylogenetic Analyses

**Figure 2:**
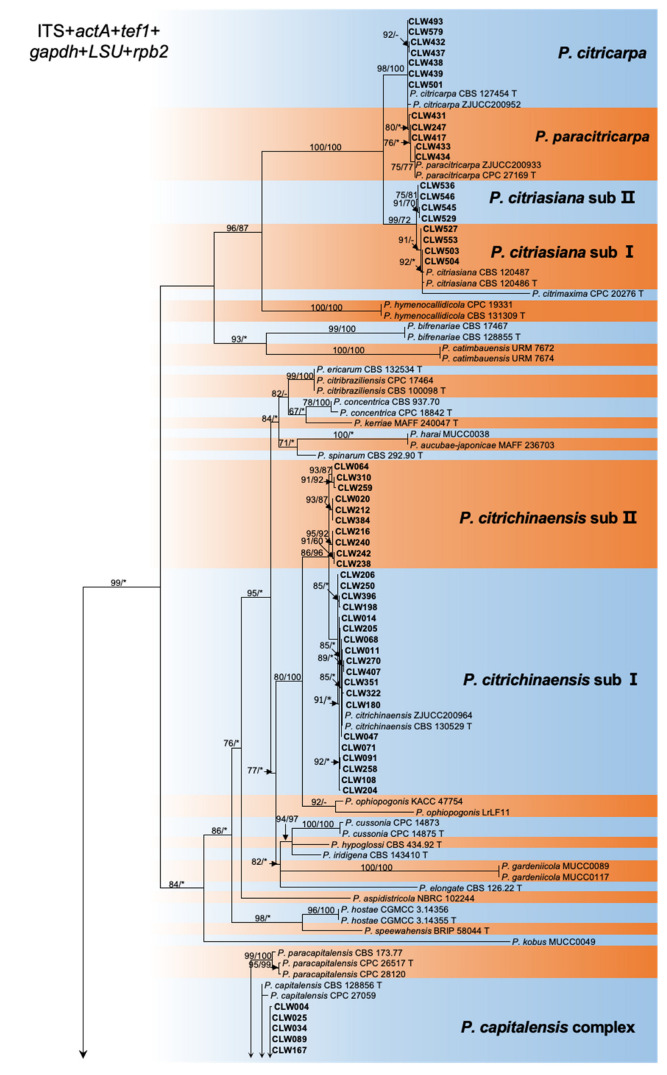

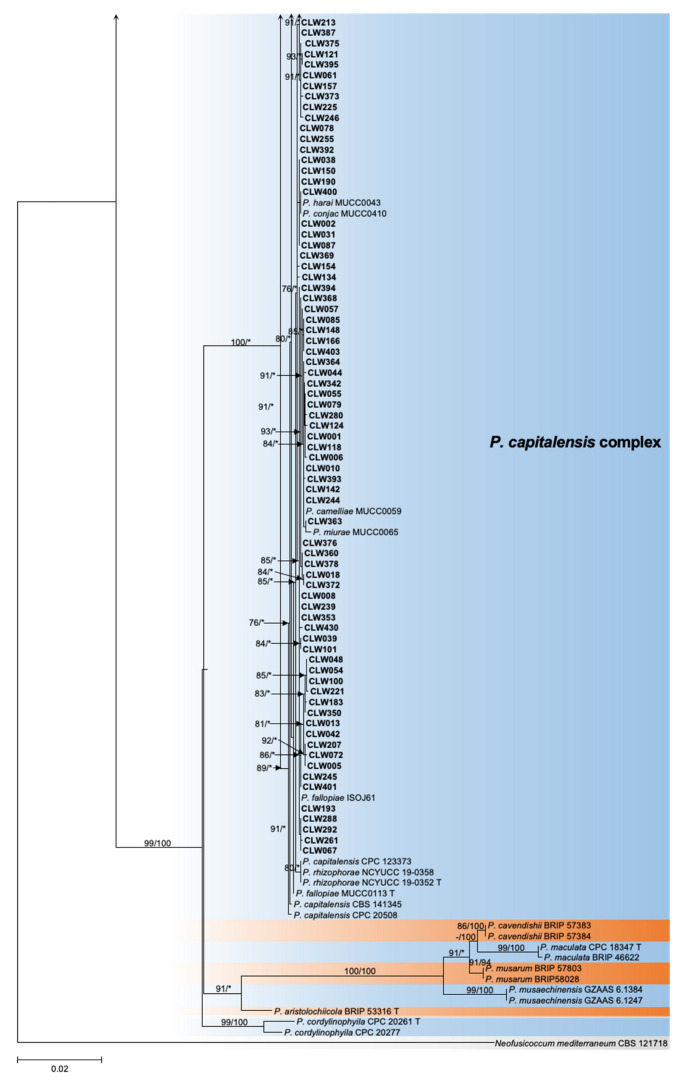
Maximum likelihood phylogeny of 194 *Phyllostica* isolates related to citrus. The tree was built using concatenated sequences of ITS, *actA*, *tef1*, *gapdh*, *LSU*, and *rpb2*. Isolates sequenced in this study are labeled in bold font. Bootstrap support values ≥ 60% for ML and MP are shown on branches as ML/MP, whereas bootstrap values < 60% or absent are marked with ‘-’, or ‘*’, respectively. Ex-type isolates are marked with ‘T’. The trees were rooted with *Neofusicoccum mediterraneum* (CBS 121718).

The ITS and *tef1* loci were amplified for all 461 strains collected from 11 provinces (Table 1, Supplementary Table S1). Taking into account the ITS and *tef1* sequence type, location, and host, a total of 188 isolates were selected for further analysis of the genotype of an additional four loci, *actA*, *gapdh*, LSU, and *rpb2* (Table 1 and Table 2, Supplementary Table S1). Subsequently, 128 representative isolates combined were selected for phylogenetic analyses based on the genotypes revealed by all six loci (Table 1, Supplementary Table S1). For the datasets of the ITS, *actA*, *tef1*, *gapdh*, LSU, and *rpb2* the PHT generated a value of *p* = 0.01, and, consequently, the sequence data for these six regions sequences were combined [47]. Multilocus data (ITS + *actA* + *tef1* + *gapdh* + LSU + *rpb2*) were composed of 193 strains of *Phyllosticta* as an ingroup and strain CBS 121718 of *Neofusicoccum mediterraneum* as an outgroup (Table 1, Supplementary Tables S1 and S2). A total of 3782 concatenated nucleotides were used in the phylogenetic analysis, viz. 1–1151 (ITS), 1152–1407 (*actA*), 1408–1671 (*tef1*), 1672–2338 (*gapdh*), 2339–3070 (LSU), and 3071–3782 (*rpb2*). Within the alignment, 2306 and 1476 positions were constant and parsimony-informative, respectively. For ML analyses, the best-fitting substitution models TIM2 + G, HKY + G, HKY + G, TrN + G, TrNef + I + G, and TrN + G were selected for ITS, *actA*, *tef1*, *gapdh*, LSU, and *rpb2*, respectively, while TIM2 + I + G and TIM2 + G were selected for the two combined datasets (Supplementary Table S3, Figure 2, Supplementary Figures S2–S7). The aligned sequences for each locus and the combined sequences of six loci were deposited in TreeBASE (http://purl.org/phylo/treebase/phylows/study/TB2:S29870; accessed on 12 December 2022). Parameters and statistics for ML and MP analyses are presented in Supplementary Table S3. For each of the eight datasets, MP and ML analyses resulted in trees with generally consistent topologies among taxa (Figure 2, Supplementary Figures S2–S7).

The 128 strains selected for phylogenetic analyses were assigned to five clusters in the phylogeny inferred from all six loci (Figure 2). Four isolates (CLW529, CLW536, CLW545, CLW546) in *P. citriasiana* cluster and twelve strains (CLW122, CLW020, CLW384, CLW216, CLW240, CLW238, CLW242, CLW064, CLW310, CLW259, CLW212, CLW287) in *P. citrichinaensis* cluster formed sub-clade with variable bootstrap support (ML: 97%, MP: 85% and ML: 93%, MP: 100%) separately (Figure 2).

### 3.3. Taxonomic Status of Subclades

#### 3.3.1. Taxonomy

Based on phylogenetic analyses and culture morphological characteristics, five well-defined species were delineated on citrus in the present study. These include *P. capitalensis*, *P. citriasiana*, *P. citricarpa*, *P. citrichinaensis*, and *P. paracitricarpa*. To further determine the taxon status of the subclades in *P. citriasiana* and *P. citrichinaensis* clusters, more morphological characteristics were used to compare the subclade isolates with its clustered known species. The asexual fruiting structure of isolates in *P. citriasiana* subclade (CLW529, CLW545) and *P. citrichinaensis* subclade (CLW238, CLW242) were produced on PNA. No significant variation was observed for two isolates of the same clade. The fruiting structures of the selected four isolates on PNA are similar to other *Phyllosticta* species [2,14,15,16,17,18]. Compared with the differences among the structures for the known *Phyllosticta* specie in the same cluster, both subclades showed that morphologically, the differences were not distinct from other strains of the same cluster [16,17]. For these reasons, these strains in the subclade were still identified as *P. citriasiana* and *P. citrichinaensis*. However, to better recognize their genetic distinctiveness and the intra-specific diversity, we designated these subclades in *P. citriasiana* and *P. citrichinaensis* as subclades I and II, separately within each of the two species. Morphological features recorded in this study, including the size of conidia, spermatia, and the optimal growth temperature on three different media, were described below.

#### 3.3.2. Morphological Characteristics

**Figure 3:**
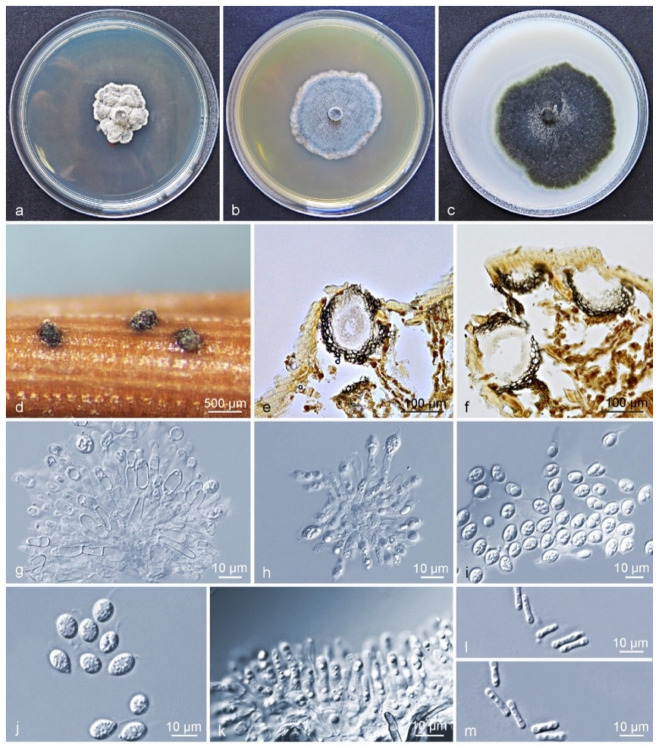
Characteristics of *Phyllosticta citriasiana* subclade II. (**a**–**c**) Living culture after 10 d on PDA, MEA, and OA (front). (**d**) Conidiomata formed on pine needle culture; (**e**,**f**) Longitudinal section through conidiomata; (**g**,**h**) conidiogenous cells and developing conidia; (**i**,**j**) conidia with mucoid sheaths and apical appendages; (**k**) spermatial cells giving rise to spermatia; (**l**,**m**) spermatia. Scale bars: (**d**) = 500 μm; (**e**,**f**) = 100 μm; (**g**–**m**) = 10 μm.

Culture characteristics (CLW529): Colonies on PDA flat white when young, turning leaden-grey and with an irregular bulge after 4–5 d, white-grey at an uneven margin. On MEA, colonies grey-white when young, gradually becoming greenish after 3 d, flat and rather regular, with white hyphae at the margin. On OA flat, greenish to grey when young, turning olivaceous-black after 3 d, irregular, with entire to feathery margin, and leaden-black underneath (Figure 3a–c). After 12 d in the dark, the optimum growth occurred at 27 °C (24 mm) on PDA, 30 °C (36 mm) on MEA, and 27 °C (55 mm) on OA. No growth was observed at 9–15 °C and 39 °C on PDA, at 9–18 °C and 39 °C on MEA, whereas growth was observed on OA at all tested temperatures. No ascostromata were observed on the inoculated PNA. Pycnidia (on PNA) immersed to erumpent, globose, subglobose to ellipsoidal. Exuded spore masses that were grey-white and opaque. Pycnidia 139–668 × 63–290 µm (Figure 3d); pycnidial wall consisting of several layers, outer wall brown to black-brown. Thickened cells tissue textura angularis to globularis; inner wall consisting of one to two pale brown cell layers, and becoming hyaline toward the interior, textura angularis (Figure 3e,f). Conidiophores subcylindrical to ampulliform, reduced to conidiogenous cells or branched from a supporting basal cell, 6–28 × 2–6 µm. Conidiogenous cells terminal, subcylindrical to ampulliform or doliiform, hyaline, smooth, coated in a mucoid layer, and proliferating once-to-several times percurrently near apex (Figure 3g,h). Conidia (8–) 9–11 (–13) × (6–) 7–9 µm, solitary, hyaline, aseptate, thin and smooth-walled, coarsely guttulate, ellipsoidal to obovoid, tapering toward a narrowly truncate base, enclosed in a thin sheath, around 1–2 µm thick, and bearing a hyaline, mucoid apical appendage, (3–) 5–9 (–16) × 1–2 µm, long pigtail, straight to flexible, unbranched, tapering towards an acutely rounded tip (Figure 3g–j). Spermatia forming in conidial conidiomata, hyaline, bacilli-form to somewhat ellipsoid, (5–) 6–7 (–8) × 1–2 µm (Figure 3k–m).

Notes: *Phyllosticta citriasiana* strains in subclade I and II were all isolated from *C. maxima* fruits in China. Between subclade I and subclade II, ten base position variations were observed within the alignment of six gene regions (Supplementary Table S5). Four fixed nucleotide changes were observed over 778 nucleotides (identity of 99.4%) for ITS; LSU contained one fixed nucleotide change over nucleotides (identity of 99.8%) whereas variations but no fixed difference was observed for *actA*, *tef1*, *gapdh*, and *rpb2* genes (Supplementary Table S5). For morphological characteristics, subclade II differs from subclade I in having larger conidiomata and conidiogenous cells, and shorter conidia, longer spermatia. The optimum temperature for growth on OA occurred at 27 °C for subclade II, whereas for the subclade I this was at 30 °C (Supplementary Table S4). However, these differences are not easily distinguished as interspecies differences. Therefore, the subclades of *P. citriasiana* reflect an intraspecific variation.

**Figure 4:**
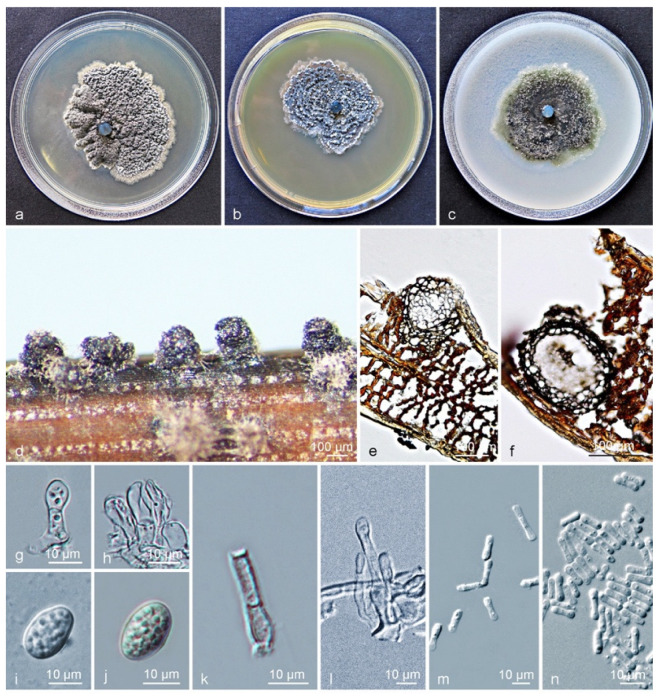
Characteristics of *Phyllosticta citrichinaensis* subclade II. (**a**–**c**) Living culture after 10 d on PDA, MEA, and OA (front). (**d**) Conidiomata formed on pine needle agar; (**e**,**f**) longitudinal section through conidiomata; (**g**,**h**) conidiogenous cells and developing conidia; (**i**,**j**) conidia with mucoid sheaths and apical appendages; (**k**,**l**) spermatial cells giving rise to spermatia; (**m**,**n**) spermatia. Scale bars: (**d**–**f**) = 100 μm; (**g**–**n**) = 10 μm.

Culture characteristics (CLW238): Colonies on PDA flat, grey-white when young, turning leaden-grey and with an irregular bulge after 4–5 d, white at the uneven margin. On MEA, colonies were grey-white when young, gradually becoming black after 3 d, granular ridges forming a concentric circle, with white hyphae at the margin. On OA, leaves were greenish to grey when young, turning olivaceous-black after 3 days, irregular, with entire-to-feathery grey-white margin, and leaden-black underneath (Figure 4a–c). After 12 d in the dark, the optimum growth occurred at 24 °C (49 mm) on PDA, 27 °C (37 mm) on MEA, and 24 °C (46 mm) on OA. No growth was observed at 9 °C and 36–39 °C on PDA, or at 9–12 °C and 36–39 °C on MEA, whereas growth on OA was observed at all tested temperatures. No ascostromata were observed on the inoculated PNA. *Pycnidia* (on PNA) erumpent to sub-immersed, subglobose to ellipsoidal (Figure 4d). Pycnidia 94–337 × 87–249 µm; pycnidial wall consisting of several layers, outer wall brown to black-brown. Thickened cells tissue *textura angularis* to globularis; inner wall consisting of one to two pale brown cell layers, and becoming hyaline toward interior, *textura angularis* (Figure 4e,f). *Conidiophores* subcylindrical to ampulliform, reduced to conidiogenous cells or branched from a supporting basal cell, 7–23 × 2–5 µm. *Conidiogenous cells* terminal, subcylindrical to ampulliform or doliiform, hyaline, smooth, coated in a mucoid layer, proliferating once to several times percurrently near the apex (Figure 4g,h). *Conidia* (8–) 10–12 × (6–) 7 (–9) µm, solitary, hyaline, aseptate, thin and smooth-walled, coarsely guttulate, ellipsoidal to obovoid, tapering toward a narrowly truncate base, sheath absent (Figure 4g–j). *Spermatia* forming in conidial conidiomata, hyaline, bacilliform to somewhat ellipsoid, (4–) 6–7 (–10) × 1–2 µm, and fewer spores observed when it formed in large numbers (Figure 4k–n).

Notes: Between subclade I and subclade II clusters of *Phyllosticta citrichinaensis*, a total of twenty-two base position variations were observed within the alignment of six loci of *P. citrichinaensis*. There were six fixed-nucleotide changes over 1151 nucleotides (identity of 98.7%) for ITS; whereas variations but no fixed difference was observed for *actA*, *tef1*, *gapdh*, LSU, and *rpb2* genes (Supplementary Table S6). For morphological characteristics, subclade II almost has no difference from subclade I, in the size and shape of conidiomata, conidiogenous cells, conidia, or spermatia. The optimum temperature for growth on PDA occurred at 24 °C for both subclades (Supplementary Table S4). Despite the divergence in phylogenetic trees, no significant morphological differences were detected. Therefore, the subclades of *P. citrichinaensis* reflect an intraspecific variation.

### 3.4. Distribution of Five Phyllosticta Species in This Study

**Figure 5:**
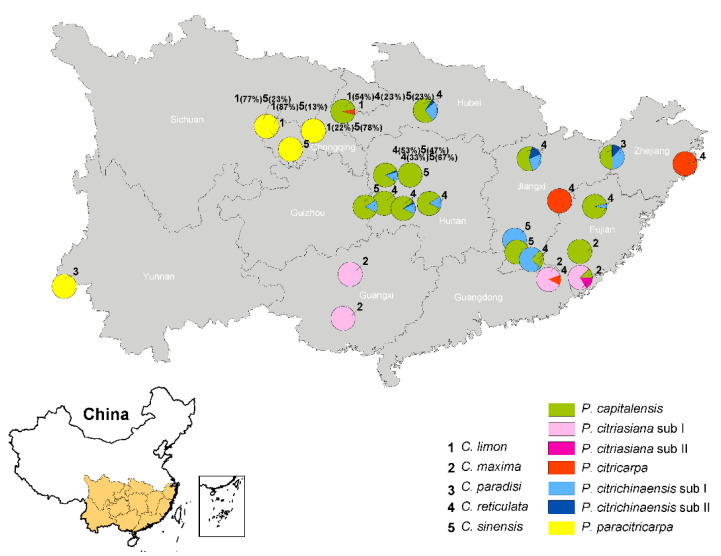
*Phyllosticta* species were detected from citrus plantations in 11 provinces of China. On the map, different species are represented by different colors with the relative proportions of each color correspond to their relative frequencies within each plantation. Numbers 1–5 represent five host citrus species, and percentages represent the proportion of isolates obtained from locations indicated on the host.

According to the phylogenetic analyses and morphological comparisons of the 461 isolates obtained from citrus in 11 provinces in southern China, five species were identified. In terms of their geographical distributions, 256 *P. capitalensis* isolates were collected from 15 plantations in eight provinces; 54 *P. citriasiana* isolates were from four plantations in three provinces; 37 *P. citricarpa* isolates were from four plantations in four provinces; 76 *P. citrichinaensis* strains were obtained from ten plantations in six provinces; and 38 *P. paracitricarpa* individuals were from four plantations of three provinces (Figure 5). In terms of host origin, *P. capitalensis* was isolated from all five *Citrus* spp. in the same or different growing areas (Figure 5); *P. citriasiana* was from *C. maxima*; *P. citricarpa* was mainly from *C. reticulata*, except one from *C. limon*; *P. citrichinaensis* was from *C. paradisi*, *C. reticulata*, *C. sinensis*; and *P. paracitricarpa* was isolated from *C. limon* and *C. sinensis* (Figure 5). The geographic and host distributions of the five species are shown in Figure 5.

### 3.5. Population Genetic Analyses

**Figure 6:**
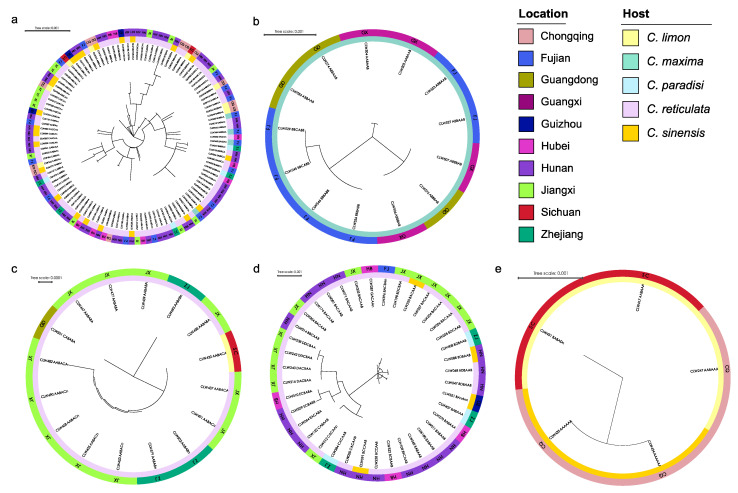
A maximum likelihood phylogenetic tree for each of five *Phyllosticta* species based on sequences at six loci in this study. (**a**) *P. capitalensis*, (**b**) *P. citriasiana*, (**c**) *P. citricarpa*, (**d**) *P. citrichinaensis*, (**e**) *P. paracitricarpa*. The colors of the outermost ring represent the sampling province and the inner color block represents the host tree. The annotation of the tree reflects the genotype and their distribution on location and host.

Based on the genotypes of strains for each species determined by the ITS, *actA*, *tef1*, *gapdh*, LSU, and *rpb2* sequences, sixty-one, five, five, thirteen, and three genotypes were generated for the isolates obtained from *P. capitalensis*, *P. citriasiana*, *P. citricarpa*, *P. citrichinaensis*, and *P. paracitricarpa*, respectively (Table 1 and Table 2). The relationships, geographic and host distribution of the genotyped strains of the five species were shown in Figure 6, with one phylogenetic tree for each of the species. These include 114 individuals for *P. capitalensis*, 13 for *P. citriasiana*, 16 for *P. citricarpa*, 39 for *P. citrichinaensis,* and five for *P. paracitricarpa*. Genotypically identical strains clustered on the same branch, but originating from multiple geographies or hosts, can be seen on all five trees (Figure 6). For example, the AABBAA genotype of *P. capitalensis* was distributed in Hubei, Hunan, Jiangxi, and Zhejiang provinces, and on host trees belonging to several varieties of *C. sinensis* and *C. reticulata* (Figure 6a). The ABBAAB genotype of *P. citriasiana* was isolated from Fujian, Guangdong, and Guangxi provinces (Figure 6b). The BDBAAB genotype of *P. citrichinaensis* was collected from Hunan and Zhejiang provinces, including on three host tree species *C. paradisi*, *C. reticulata*, and *C. sinensis* (Figure 6d). Genotype AABAAA of *P. paracitricarpa* was found on *C. limon* in Chongqing municipality and Sichuan province (Figure 6e).

AMOVA analyses showed no significant genetic differentiation between either geographic (i.e., provincial) or host subpopulations for *P. capitalensis*, *P. citricarpa*, and *P. citrichinaensis* (Table S7). However, for provincial subpopulations of *P. citriasiana* and host subpopulations of *P. paracitricarpa*, high Fst values of 0.529 (Guangxi vs. Fujian), 0.579 (Guangxi vs. Guangdong), and 0.571 (C. sinensis vs. C. limon) were observed, consistent with some levels of genetic differentiation (Table S7). Phylogenetic incompatibility analyses revealed evidence for recombination within *P. capitalensis* and *P. citrichinaensis* (Table 3); however, linkage equilibrium rejected the hypothesis of random recombination across most samples (*p* < 0.05) for *P. capitalensis* (P_NCC_ = 0.001, P_CC_ = 0.001), *P. citriasiana* (P_NCC_ = 0.001, P_CC_ = 0.003), and *P. citrichinaensis* (P_NCC_ = 0.001, P_CC_ = 0.001) (Table 2). Together, the combined results based on phylogenetic analyses, AMOVA, LD, and Prp revealed evidence for clonal expansion in all five species across its sampled regions and host trees.

### 3.6. Pathogenicity

**Figure 7:**
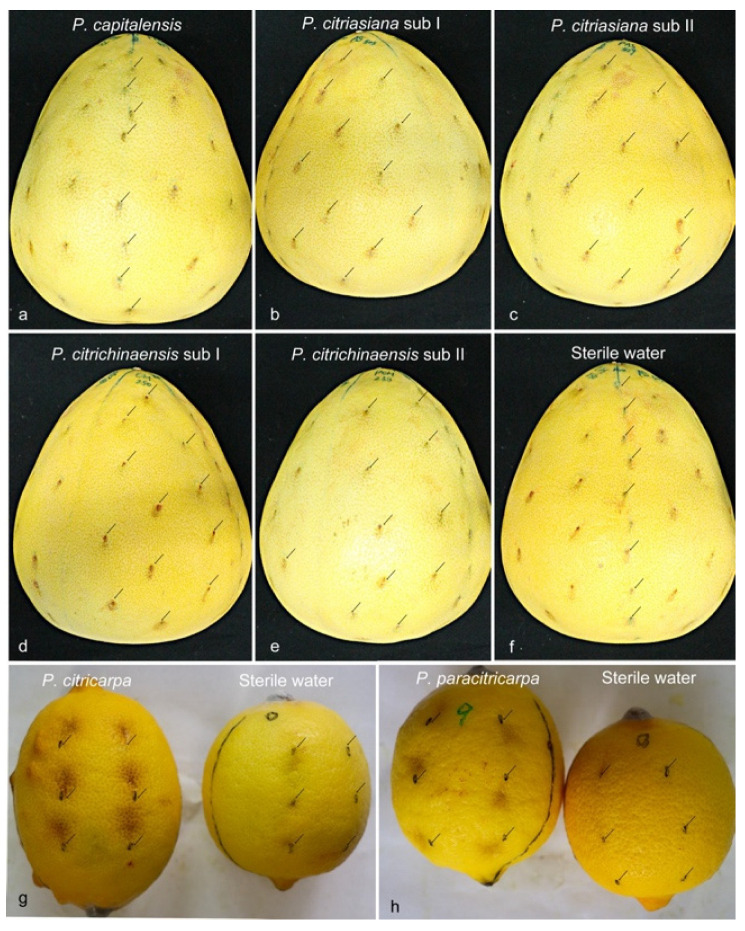
Pathogenicity assay of *P. capitalensis*, *P. citriasiana* subclade I and subclade II, *P. citrichinaensis* subclade I and subclade II on fruits of *C. maxima* cv. Guanximiyou, *P. citricarpa* and *P. paracitricarpa* on fruits of *C. limon*; (**a**–**f**) *C. maxima* cv. Guanximiyou fruits inoculated with *P. capitalensis*, *P. citriasiana* subclade I and subclade II, *P. citrichinaensis* subclade I and subclade II, and sterile water, (**g**,**h**) *C. limon* fruits inoculated *P. citricarpa* (fruit on the left), *P. paracitricarpa* (fruit on the left) and sterile water (fruit on the right). Black arrows indicate the injected positions.

A total of seven strains were selected for the pathogenicity test. One for each of the five species and one for each of the two subclades of *P. citriasiana* and *P. citrichinaensis*. Since *P. citriasiana* was only isolated from *C. maxima*, *P. capitalensis*, and *P. citrichinaensis* can be found from *C. maxima*, while *C. maxima* is resistant to *P. citricarpa* [16,17,48], for pathogenicity test, *P. capitalensis*, *P. citriasiana*, and *P. citrichinaensis* were inoculated on *C. maxima* cv. Guanximiyou, *P. citricarpa*, and *P. paracitricarpa* were injected into *C. limon* fruits. Twenty-five days after inoculation, all isolates of the inoculated species induced lesions on the inoculated points of fruits. In general, the inoculated fruits developed spots similar to those observed in the field, which were light to dark brown, and sunken in the center of the spot (Figure 7). However, a clear difference in the inoculation between the *Phyllosticta* species was observed (Figure 7 and Figure S8). *P. citricarpa* caused maximum lesions on its tested fruits (Figure 7g and Figure S8), followed by *P. paracitricarpa* (Figure 7f and Figure S8) then *P. citriasiana* (Figure 7b,c and Figure S8), *P. citrichinaensis* (Figure 7d,e and Figure S8), and *P. capitalensis* (Figure 7a and Figure S8). No significant difference in pathogenicity was found between *P. citriasiana* and *P. citrichinensis* for *C. maxima* (Figure 7a,f).

## 4. Discussion

This study reports extensive *Phyllosticta* collections from diseased citrus materials, and these strains were identified as belonging to five species, including 256 isolates identified as *P. capitalensis*, 76 as *P. citrichinaensis* (64 in subclade I, 12 in subclade II), isolated from various citrus leaves or fruits with freck or minute spots (Figure 2). Additionally, 54 isolates of *P. citriasiana* (50 in subclade I, four in subclade II) were identified from *C. maxima* fruits with typical tan spot symptoms (Figure S1), while 37 in *P. citricarpa* and 38 in *P. paracitricarpa* were isolated from *C. paradisi*, *C. limon*, *C. reticulata*, and *C. sinensis* fruits with black spot symptoms (Figure 1 and Figure S1). Together, these five species showed geographic region-biased disributions. For example, *P. citriasiana* was mainly from Guangdong, Guangxi, and Fujian provinces; *P. citricarpa* and *P. paracitricarpa* were mainly from Chongqing, Guangdong, Zhejiang, Sichuan, Jiangxi, and Sichuan provinces or municipality. Overall, the pathogen distributions largely overlap with the cultivation regions of their respective host plants, consistent with host plant and/or geographic separation playing a role in *Phyllosticta* species distributions.

*Phyllosticta capitalensis* is often reported as an endophyte or weak plant pathogen with a vast host range [15]. Recently, it was reported as the main pathogen of leaf spot on oil palm, *Camellia sinensis*, *Ricinus communis,* and fruit spot on *Psidium guajava* [49,50,51,52]. In this study, *P. capitalensis* strains were isolated from the leaves and fruits of various *Citrus* varieties with freckles or minute spots, and the pathogenicity test on fruits show slight symptoms, indicating that it might be a weak pathogen on citrus. Wang et al. [17] first described *P. citrichinaensis* on leaves and fruits with some irregular freckle spots, from Chongqing, Guangdong, Fujian, Shannxi, Sichuan, and Zhejiang provinces in China. In this study, we found that *P. citrichinaensis* strains were isolated from diseased tissues with symptoms similar to those reported but from four additional provinces Guizhou, Hubei, Hunan, and Jiangxi in China. The results of the current study support an earlier study that suggested that *P. citrichinaensis* have a wide geographic and host distribution [17]. But inoculation test in this research showed that *P. citrichinaensis* produces similar lesions as that of *P. citriasiana* on *C. maxima* fruits. From leaf freckle spot, *P. capitalensis* and *P. citrichinaensis* were the major isolates. Although no negative impact on citrus production such as defoliation or dropped fruit has been reported, these freckles or minute spots can decrease the market value of citrus fruits.

Currently, eight *Phyllosticta* species have been described on citrus worldwide. *Phyllosticta citricarpa* and *P. capitalensis* are present on all cultivated citrus species; *P. paracapitalensis* was reported from Europe and New Zealand; *P. citribraziliensis* was reported only in South America; *P. paracitricarpa* is present in Asia (China) and Europe (Greece); and *P. citrichinaensis*, *P. citriasiana*, and *P. citrimaxima* were only found in Asia [2,14,17,18]. In the current study, *P. citriasiana* and *P. citrichinaensis* contained subclades with high bootstrap supports in phylogenetic trees, similar to *P. citricarpa* and its sister species, *P. paracitricarpa*. However, no significant morphological difference was found between these subclades. Combined with the fewer fixed difference in their sequences than between *P. citriacarpa* and *P. paracitricarpa*, the speciation between *P. citriasiana* and *P. citrichinaensis* is likely ongoing and incomplete [53]. Although abundant isolates of *P. capitalensis* were obtained, *P. paracapitalensis*, which was reported in New Zealand, Italy, and Spain [2], and the endophyte *P. citribraziliensis*, reported in Brazil [14], were not discovered in this study. *Phyllosticta citrimaxima*, which is associated with tan spots of *C. maxima* fruit in Thailand [15], was not isolated from *C. maxima* in China. However, *P. citrichinaensis* was isolated from broader *Citrus* varieties and citrus planting areas than that reported by Wang et al. [17] in China (Table 1 and Table 2). Based on the species identification results, *P. citricarpa* was found on all *Citrus* species except *C. maxima*, which is consistent with the speculation that this species may not infect *C. maxima* [6]. In this study, *P. paracitricarpa* was discovered to have a wider host and geographic distribution on *Citrus* spp. in China (Table 1 and Table 2), such as *C. paradisi*, and this is also the first report of this species in Yunnan province (Table 1 and Table 2).

Based on the ITS, *actA*, *tef1*, *gapdh*, LSU, and *rpb2* sequence data, the multilocus genotype of each isolate was determined in the present study. The results indicated that the genotypic diversity of *P. capitalensis* and *P. citrichinaensis* was higher than the other three *Phyllosticta* species (Table 1 and Table 2, Supplementary Table S1). Generally, rich genetic diversity facilitates the adaptation of pathogens to their environment or host. However, based on allelic information at the six loci, the genetic diversities of the most and second-most pathogenic *P. citricarpa* and *P. paracitricarpa* were lower than those in others. Earlier studies based on microsatellite loci and whole-genome sequencing revealed rich genetic diversity within *P. citricarpa* [19,21,22]. The greater diversity observed using microsatellite loci and whole-genome sequencing that multilocus sequence typing (MLST) is not surprising because microsatellite loci evolve much faster than single nucleotide substitutions and whole-genome sequencing analyzes far more loci than MLST [54,55]. Several factors such as mating system, gene flow, and selection can all influence genetic diversity in a population [54,55]. Interestingly, though *P. citriasiana*, *P. citricarpa*, and *P. paracitricarpa* have heterothallic mating systems, no ascomata have been observed for these species in the field [21,22,24,56,57]. However, the absence of observed sexual structures does not preclude the possibility that these species may be capable of sexual outcrossing. Several population research studies have revealed that sexual recombination can occur in the field for *P. citriasiana* in China [24] and *P. citricarpa* in South America, Brazil, and Australia [21,22]. However, whether sexual reproduction occurs in *P. citricarpa* and *P. paracitricarpa* in China remains to be determined.

Our analyses revealed evidence for clonal expansion for all five species within and across the sampled regions. Similar results have been reported for others citrus fungal pathogens like *Diaporthe citri* [58] and *P. citriasiana* [24]. Although sexual spores are often considered as the primary source of dispersal and infections [56,59], clonal expansion of specific genotypes has been reported in many pathogens, leading to serious disease epidemics [53,60,61,62,63,64]. Examples of clonal expansion in fungal pathogens include the banana wilt, caused by Foc TR4 that has been reported across various regions in at least 17 countries in the world [53,61,62]. Potato late blight, responsible for the Irish Potato Famine, was caused by a single clonal lineage of *Phytophthora infestans* [53,60,65]. Similarly, a single clonal lineage of *Cryphonectria parasitica* dominated most geographic populations of chestnut across southern Europe [66]. Considering that asexual conidia are mainly dispersed by raindrops over short distances, the clonal spread of adapted genotypes across the five *Phyllosticta* species in different plantation areas was likely facilitated by human-mediated dispersal, such as human trade, travel, and germplasm exchange, as have been suggested for many fungal pathogens [67].

The present study provides a systematic method for strain selection and identification of *Phyllosticta* species on various types of spot symptoms of citrus. The results revealed a high level of interspecific and intraspecific diversity of *Phyllosticta* associated with citrus in China (Table 1, Supplementary Table S1). The most commercially harmful *Phyllosticta* disease to the citrus industry in China is black spot caused by *P. citricarpa* and *P. paracitricarpa*. Our results suggest that targeted markers should be developed to monitor the prevalence and spread of clonal genotypes in these species [53,64]. In addition, the highly prevalent genotypes should be further evaluated for their susceptibilities to fungicides and for their genomic features [60,61]. Accurate monitoring of pathogen species, genotypes, and their susceptibilities to agricultural fungicides, both spatially and temporarily, is needed to help develop better management strategies for the prevention and control of citrus black spots and similar diseases in citrus plantations [67].

## Figures and Tables

**Table 1:** Information on *Phyllosticta* isolates collected from citrus in China and their genotype statistics based on geographical origin.

Species	Location	Iso. Num.	Genotype Based on DNA Sequences at Six Loci ^1^
*P. capitalensis*	Chongqing	13	AABBA-, ABBBAA, ABBCA-, BABBAA, BBBBA-, BBBCA-, BBBFAA, CABBA-, CABGA-
	Fujian	19	AABBAB, AABCA-, ABBBAA, ABBCAA, BABBAA, BABBC-, BABBCA, BABCC-, BABDA-, BABEA-, B-BBAA, CABBA-, CABDA-, C-BFA-, NABBAA
	Guizhou	4	ABBC-A, ACBCA-, CABCA-
	Hubei	49	AABBAA, AABBA-, AAB-AA, BABBCA, BAB---, DABBCA, EBBFAA
	Hunan	140	AABBAA, AABCAA, ABBCA-, ACBBAA, ACBCBA, BABBA-, BABBAA, BABBC-, BABBCA, BABCA-, BABCC-, BABCCA, BBBBCA, BCBBA-, CABBAA, CABCA-, CABCAA, CABDAA, CABEAA, CABFA-, CACCA-, EABCA-, EBBBAA, EBBCAA, ECBCA-, ECBCAA, FBBCAA, GABBC-, HABBA-, CBBAA, JABCAA, KABBCA, LABBAA
	Jiangxi	24	AABBAA, ABBCAA, ABBHAA, BABCCA, BABBC-, BCBBA-, CABBA-, CABCA-, ECBBAA
	Sichuan	2	BBBFAA, MBBCCA
	Zhejiang	5	AABBAA, AABCAA, BABBCA, BBBBC-, CABED-
*P. citriasiana*	Fujian	21	ABBAAB, BBBABB, BBCABB
	Guangdong	13	ABBAAB, ABBBAB
	Guangxi	20	AABAAB, ABBBAB
*P. citricarpa*	Guangdong	1	CABABA
	Jiangxi	11	AABABA, AABACA
	Sichuan	1	AABACA
	Zhejiang	24	AABAB-
*P. citrichinaensis*	Fujian	1	BACBA-
	Guizhou	1	BA-A--
	Hubei	17	BCBAAB, ECBABA, GACAA-
	Hunan	35	BABAAB, BACAAB, BBCAAB, BCCAAB, BDBAAB, BFCAAB, CABAAB, CACAAB, EACABA, ECBABB
	Jiangxi	18	BACAAA, BACAAB. BDCAAB, BDCBAA, CACAA-, DACBAA, DDCBAA
	Zhejiang	4	BABAAA, BDBAAB, CACAAB
*P. paracitricarpa*	Chongqing	19	AAAAAA, AABAAA
	Sichuan	18	AABAAA, BABAD-
	Yunnan	1	--A---

^1^ Genotypes were determined based on sequence alignment to their type strains, the type strain was designated as AAAAAA (ITS, *actA*, *tef1*, *gapdh*, *LSU*, *rpb2*), “-” means the sequence at this locus is absent.

**Table 2:** Information of *Phyllosticta* isolates collected from citrus in China and their genotype statistics based on isolated host tree species.

	Host	Iso. Num.	Genotype Based on DNA Sequences at Six Loci ^1^
*P. capitalensis*	*C. limon*	9	ABBBAA, ABBCA-, BABBAA, BBBBA-, BBBFAA, CABGA-, MBBCCA
	*C. maxima*	5	BABBC-, BABDA-, BABEA-, B-BBAA, C-----
	*C. paradisi*	4	AABCAA, BABBCA, BBBBC-, CABED-
	*C. reticulata*	199	AAB-AA, AABB--, AABBA-, AABBAA, AABBAB, AABCA-, AABCAA, ABBBAA, ABBCAA, ABBHAA, ACBBAA, ACBCBA, BAB---, BABBA-, BABBAA, BABBC-, BABBCA, BABCA-, BABCC-, BABCCA, BBBBCA, BBBCA-, BBBFAA, BCBBA-, CABBA-, CABBAA, CABCA-, CABCAA, CABDA-, CABDAA, CABEAA, CABFA-, C-BFA-, DABBCA, EABCA-, EBBBAA, EBBCAA, EBBFAA, ECBBAA, ECBCA-, ECBCAA, GABBC-, HABBA-, ICBBAA, JABCAA, LABBAA, NABBAA
	*C. sinensis*	39	AABBA-, AABBAA, ABBBAA, ABBCA-, ABBC-A, ACBCA-, BABBC-, BBBFAA, BCBBA-, CABBA-, CABCA-, CABCAA, CACCA-, ECBCAA, FBBCAA, KABBCA, LABBAA
*P. citriasiana*	*C. maxima*	54	AABAAB, ABBAAB, ABBBAB, BBBABB, BBCABB
*P. citricarpa*	*C. reticulata*	37	AABABA, AABACA, CABABA
*P. citrichinaensis*	*C. paradisi*	4	BABAAA, BDBAAB, CACAAB
	*C. reticulata*	65	BABAAA, BABAAB, BACAAA, BACAAB, BACBA-, BBCAAB, BCBAAB, BCCAAB, BDBAAB, BDCAAB, BDCBAA, BFCAAB, CABAAB, CACAA-, CACAAB, DACBAA, DDCBAA, EACABA, ECBABA, ECBABB, GACAA-
	*C. sinensis*	7	BA-A--, BACAAA, BCCAAB, BDBAAB
*P. paracitricarpa*	C. limon	19	AABAAA, BABAD-
	*C. paradisi*	1	--A---
	*C. sinensis*	18	AAAAAA

^1^ Genotypes were determined based on sequence alignment to their type strains, the type strain was designated as AAAAAA (ITS, *actA*, *tef1*, *gapdh*, *LSU*, *rpb2*), “-” means the sequence at this locus is absent.

**Table 3:** Linkage disequilibrium and recombination analyses for five Phyllosticta species based on allelic information at six loci in this study.

Scale	Non-Clone-Corrected Data						Clone-Corrected Data					
	N ^1^	IA ^2^	Rd ^3^	*p* ^4^	Prp ^5^	*p* ^6^	N ^1^	IA ^2^	Rd ^3^	*p* ^4^	Prp ^5^	*p* ^6^
*P. capitalensis*	114	1.010	0.046	0.001	0.947	0.001	59	1.040	0.044	0.001	0.947	0.001
*P. citriasiana*	13	1.950	0.326	0.001	1.000	0.001	5	1.400	0.234	0.003	1.000	0.002
*P. citricarpa*	16	0.000	0.000	1.000	1.000	1.000	3	-0.500	-0.500	1.000	1.000	1.000
*P. citrichinaensis*	37	4.820	0.264	0.001	0.865	0.001	21	3.520	0.189	0.001	0.865	0.001
*P. paracitricarpa*	5	1.200	0.300	0.021	1.000	0.094	3	0.400	0.100	0.377	1.000	1.000

^1^ N: number of isolates; ^2^ IA: index of association; ^3,^ rd: standard index of association; ^4^ *p* values of IA and rd, calculated in package poppr with 999 permutations; ^5^ Prp: Proportion of compatible pairs of loci; ^6^ *p* values of Prp, calculated in Multilocus 1.3b with 1000 permutations.

## Data Availability

Not applicable.

## Supplementary Materials

The following supporting information can be downloaded at: https://www.mdpi.com/article/10.3390/jof9040449/s1, Figure S1: Symptoms of citrus fruit spots from which the *Phyllostitca* isolates were obtained; Figures S2–S7: Maximum likelihood phylogeny of *Phyllostica* isolates related to citrus. S2. 194 isolates of ITS tree, S3. 183 isolates of *actA* tree, S4. 176 isolates of *tef1* tree, S5. 166 isolates of *gapdh* tree, S6. 164 isolates of LSU tree, S7. 116 isolates of *rpb2* tree; Figure S8. Column chart indicating the average lesion area produced each isolate of *Phyllosticta* spp. Table S1: Isolates information sequenced in this study; Table S2: GenBank Accession number of the isolates used for phylogenetic analysis in this study. Table S3: Datasets used and statistics resulting from phylogenetic analyses. Table S4: Comparison of morphology of two novel *Phyllosticta* species and their related sister species. Table S5: Nucleotide differences observed among *P. paracitriasiana* and *P. citriasiana* isolates used in this study. Table S6: Nucleotide differences observed among *P. paracitrichinaensis* and *P. citrichinaensis* isolates used in this study. Table S7: Fst values among provincial or/and host subpopulations of five *Phyllostitca* spp. in China.
