# Supplementary material for: Clonal Expansion in Multiple Phyllosticta Species Causing Citrus Black Spot or Similar Symptoms in China
Source: J Fungi (Basel). 2023 Apr 6;9(4):449. doi: 10.3390/jof9040449 (PMC10145914; doi:10.3390/jof9040449)
Supplement: Supplementary file 1 [file jof-09-00449-s001.zip › Supplementary Figures S1-S8.pdf]

Journal of *Fungi*

MDPI

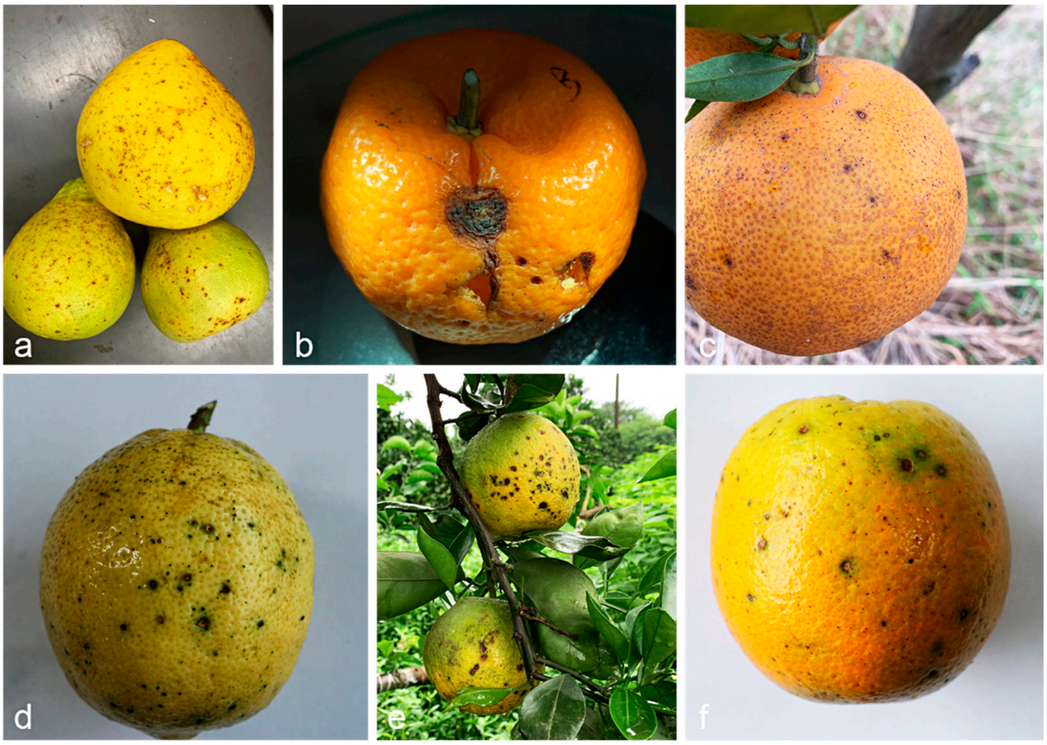

**Figure S1** Symptoms of citrus fruit spots from which the *Phyllostitca isolates* were obtained. a *C. maxima* fruits, b *C. reticulata* cv. Nangfengmiju, c *C. reticulata* cv. Unshiu, d *C. limon*, e *C. sinensis* cv. Navel, f *C. sinensis* cv. Valencia.

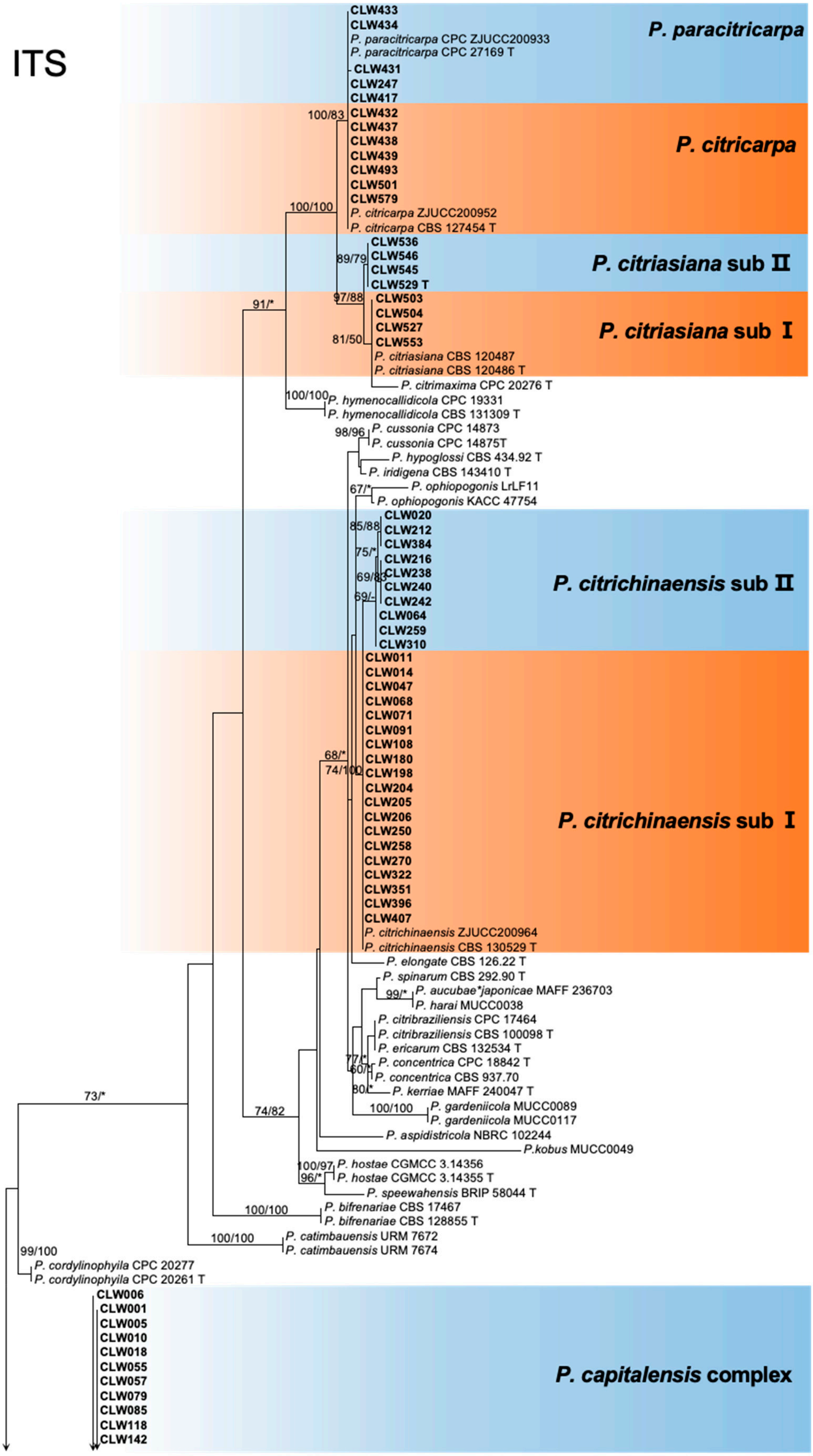


Next page continue.

Continue

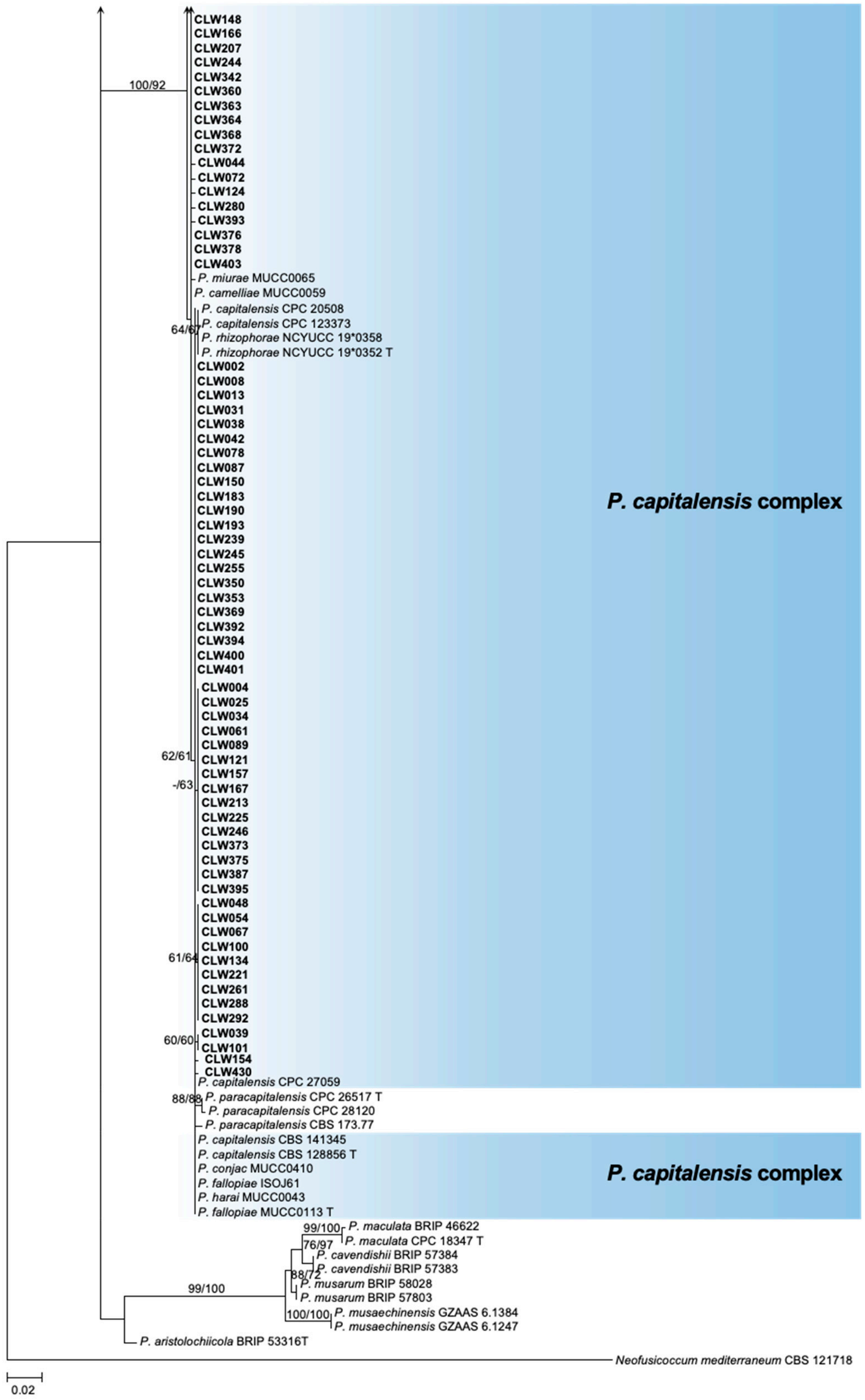


**Figure S2.** Maximum likelihood phylogeny of *Phyllostica* isolates related to citrus. 194 isolates of ITS tree.

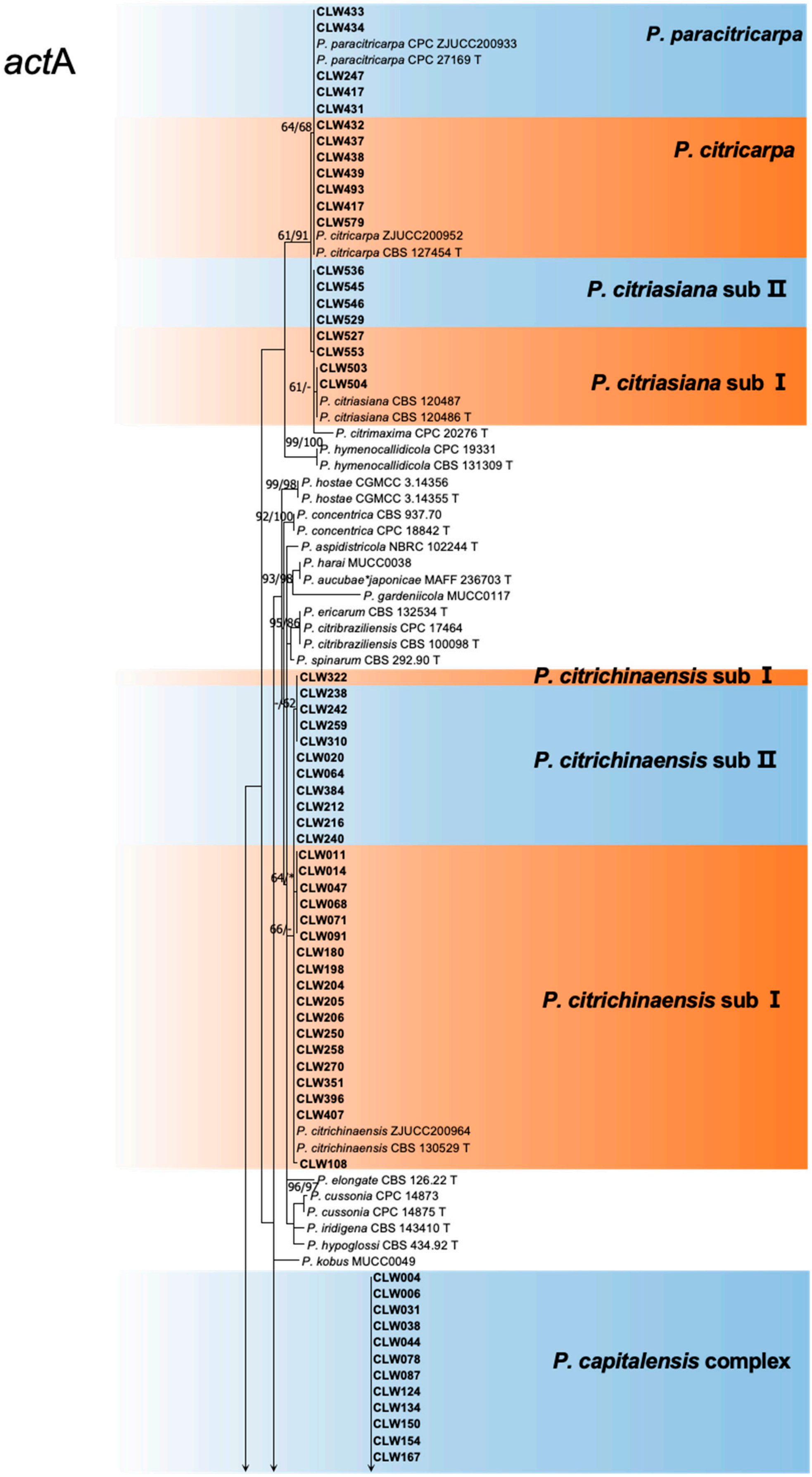


Next page continue.

Continue.

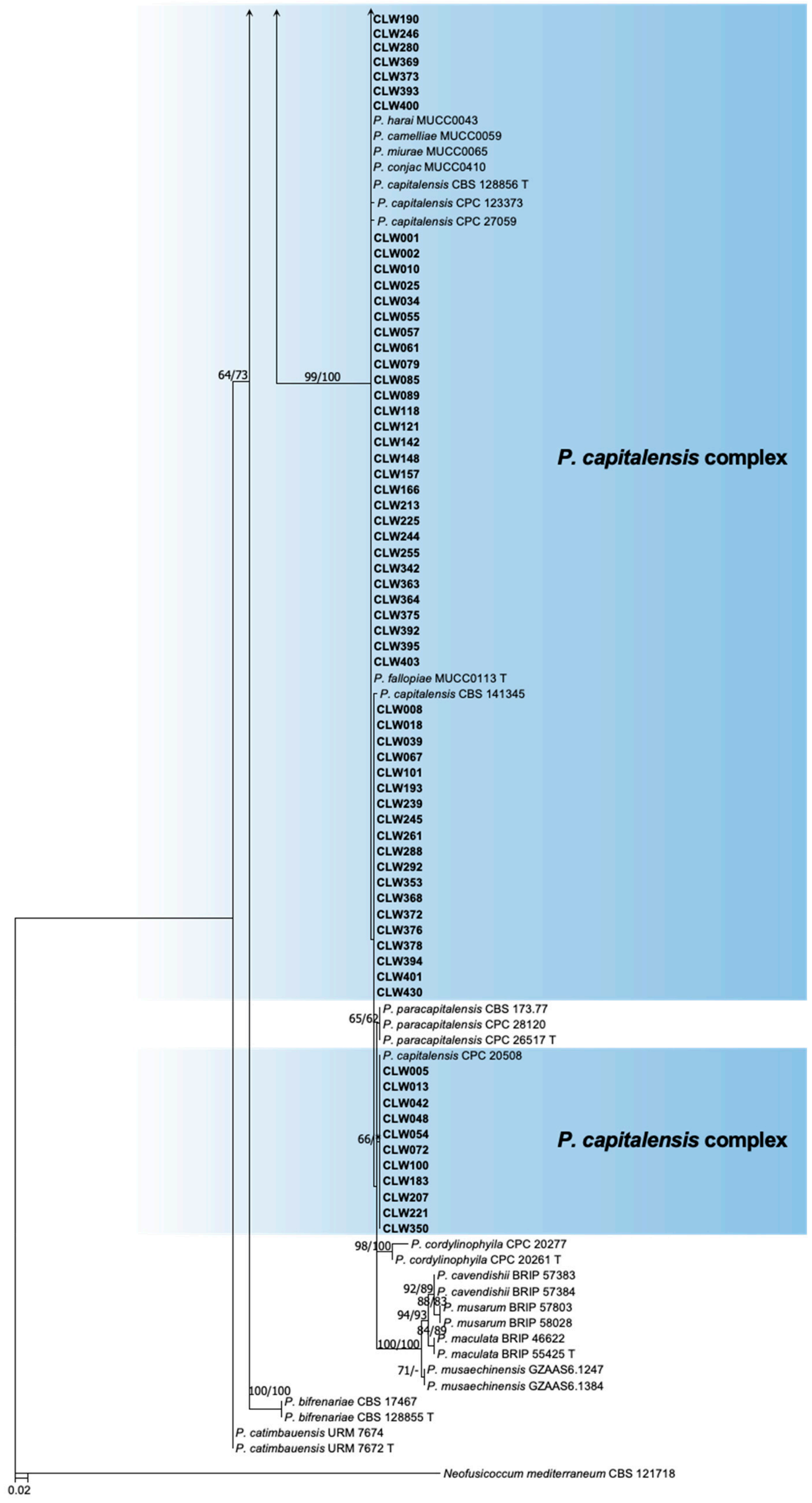


**Figure S3.** Maximum likelihood phylogeny of *Phyllostica* isolates related to citrus. 183 isolates of *actA* tree.

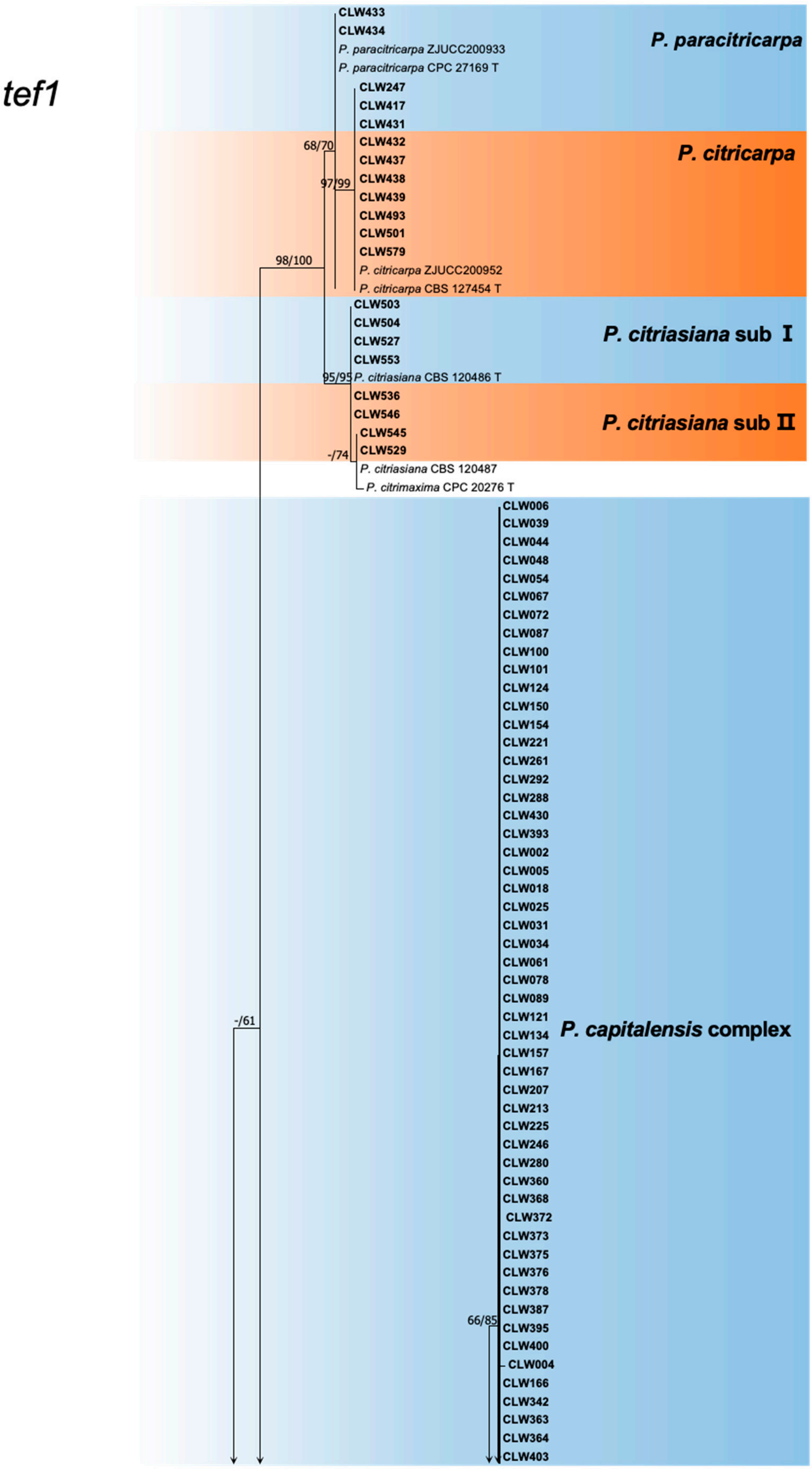


Next page continue.

Continue.

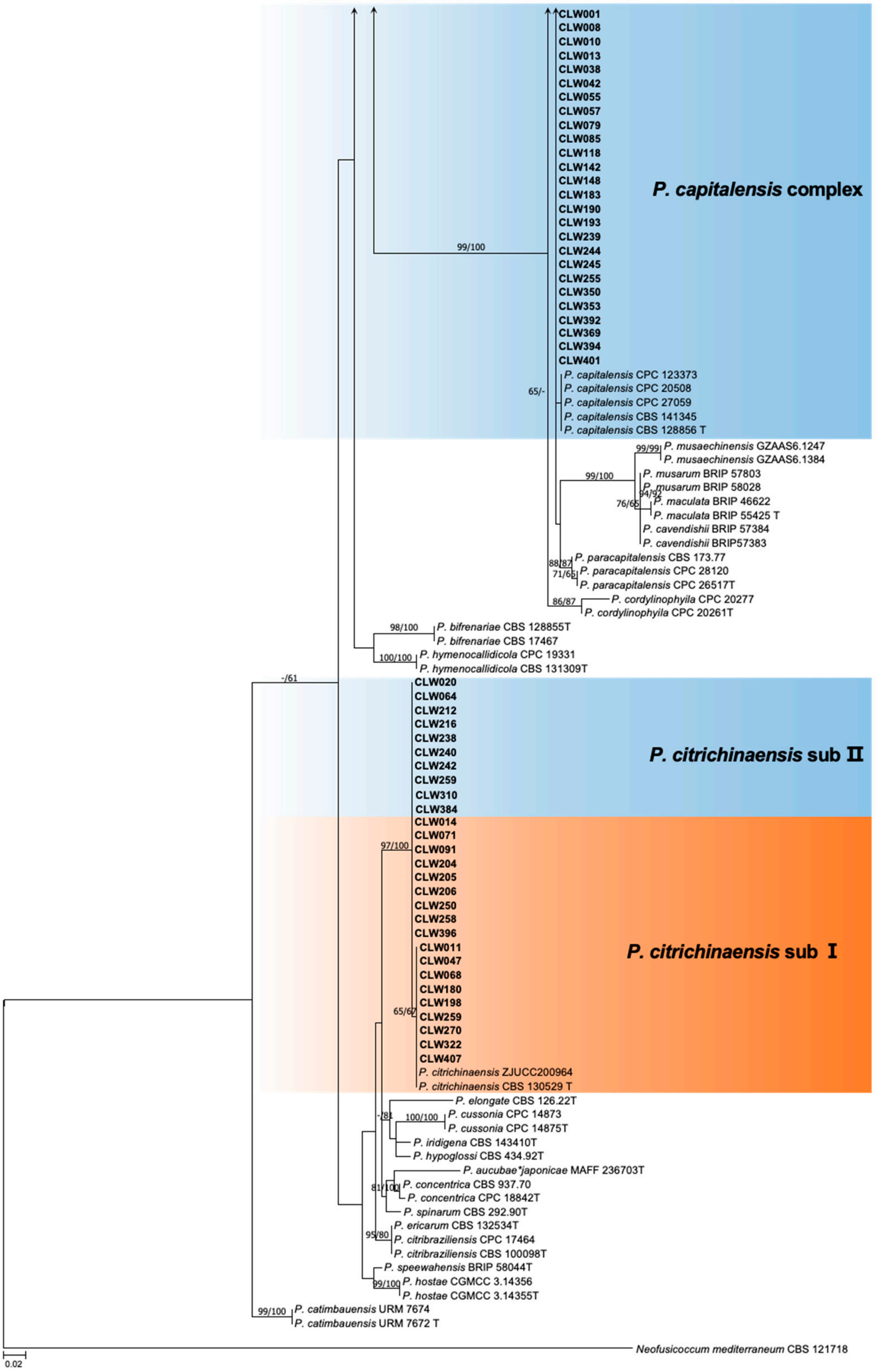


**Figure S4.** Maximum likelihood phylogeny of *Phyllostica* isolates related to citrus. 176 isolates of *tef1* tree.

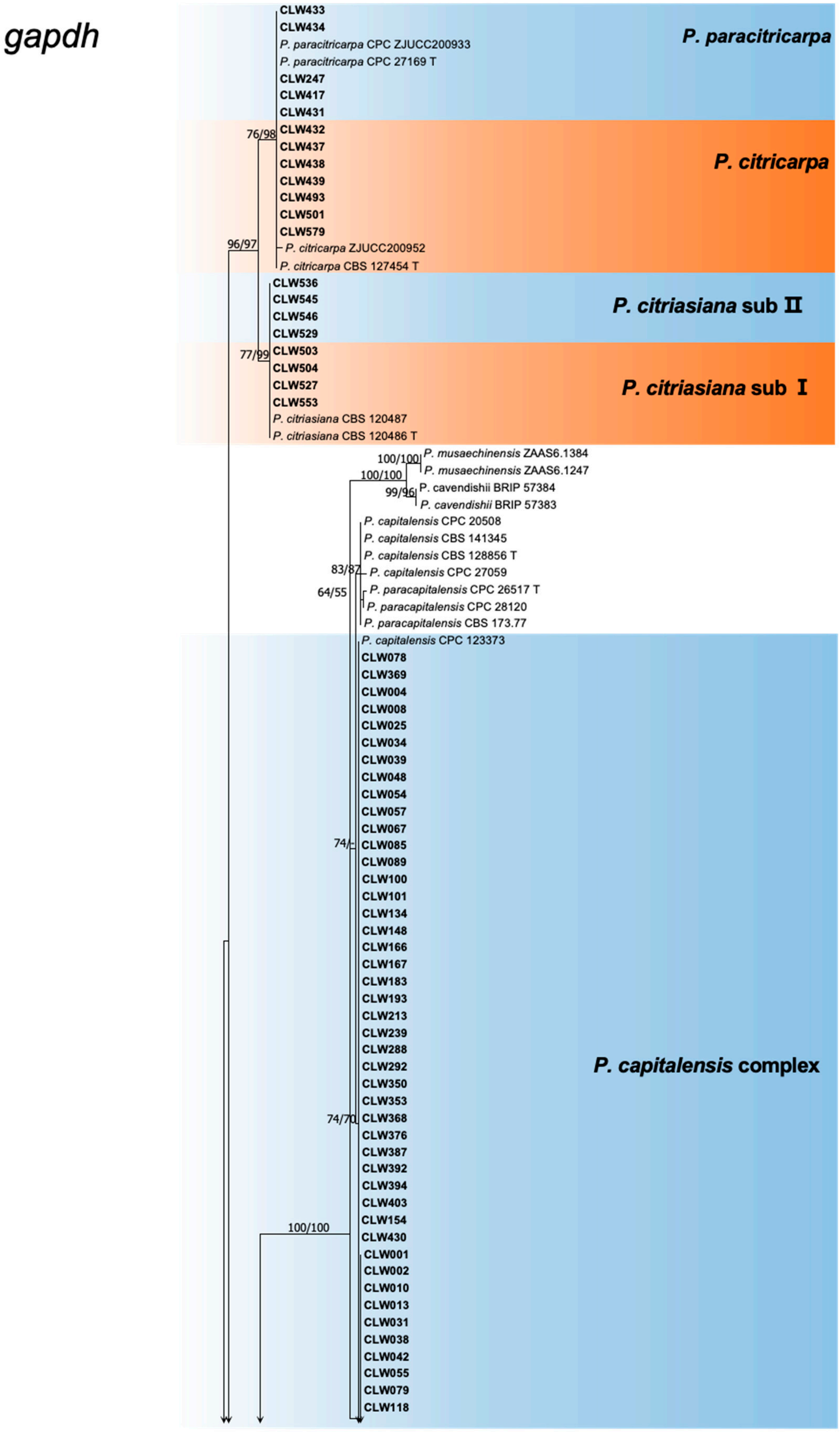


Next page continue.

Continue.

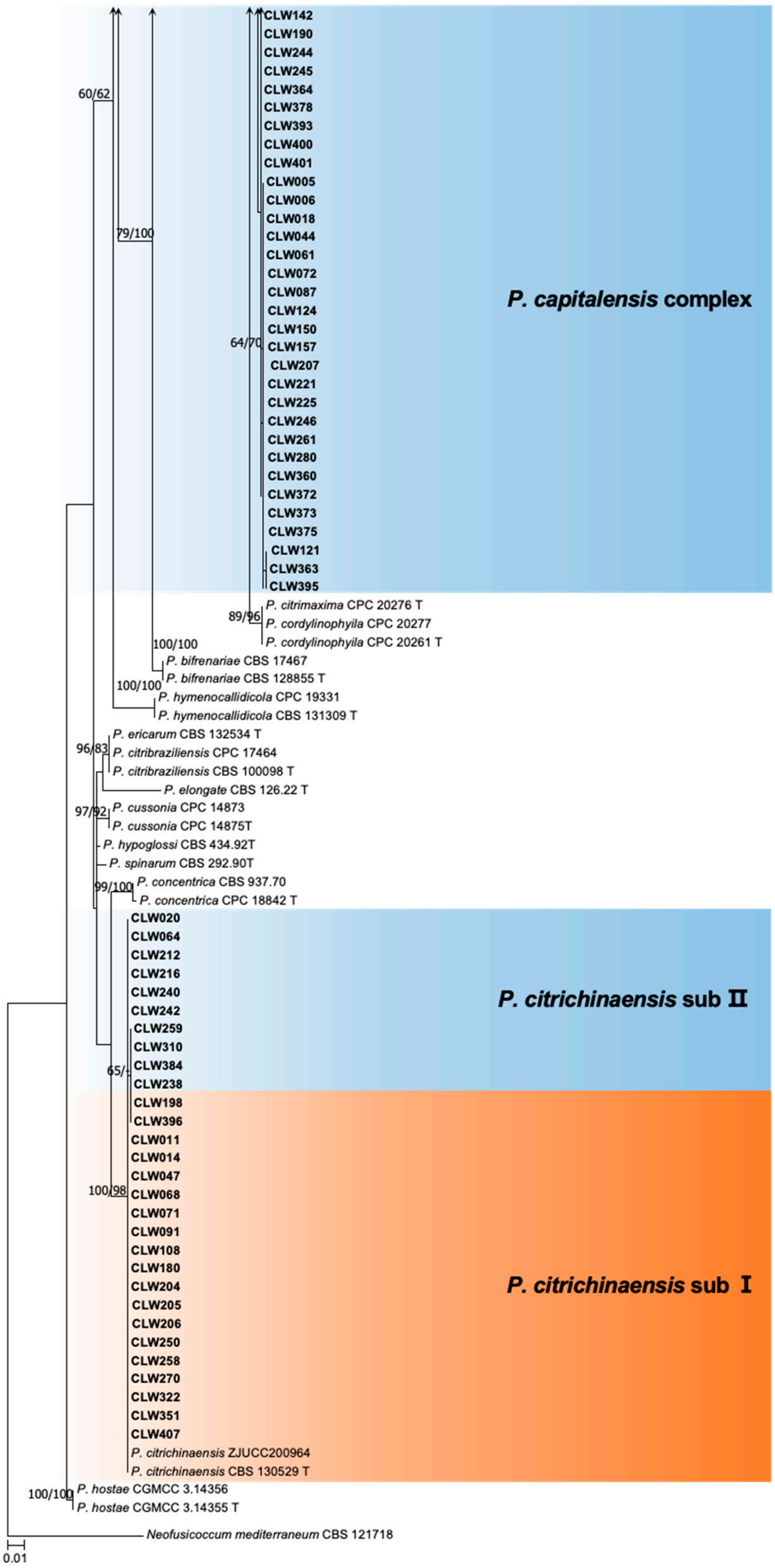


**Figure S5** Maximum likelihood phylogeny of *Phyllostica* isolates related to citrus. 166 isolates of *gapdh* tree.

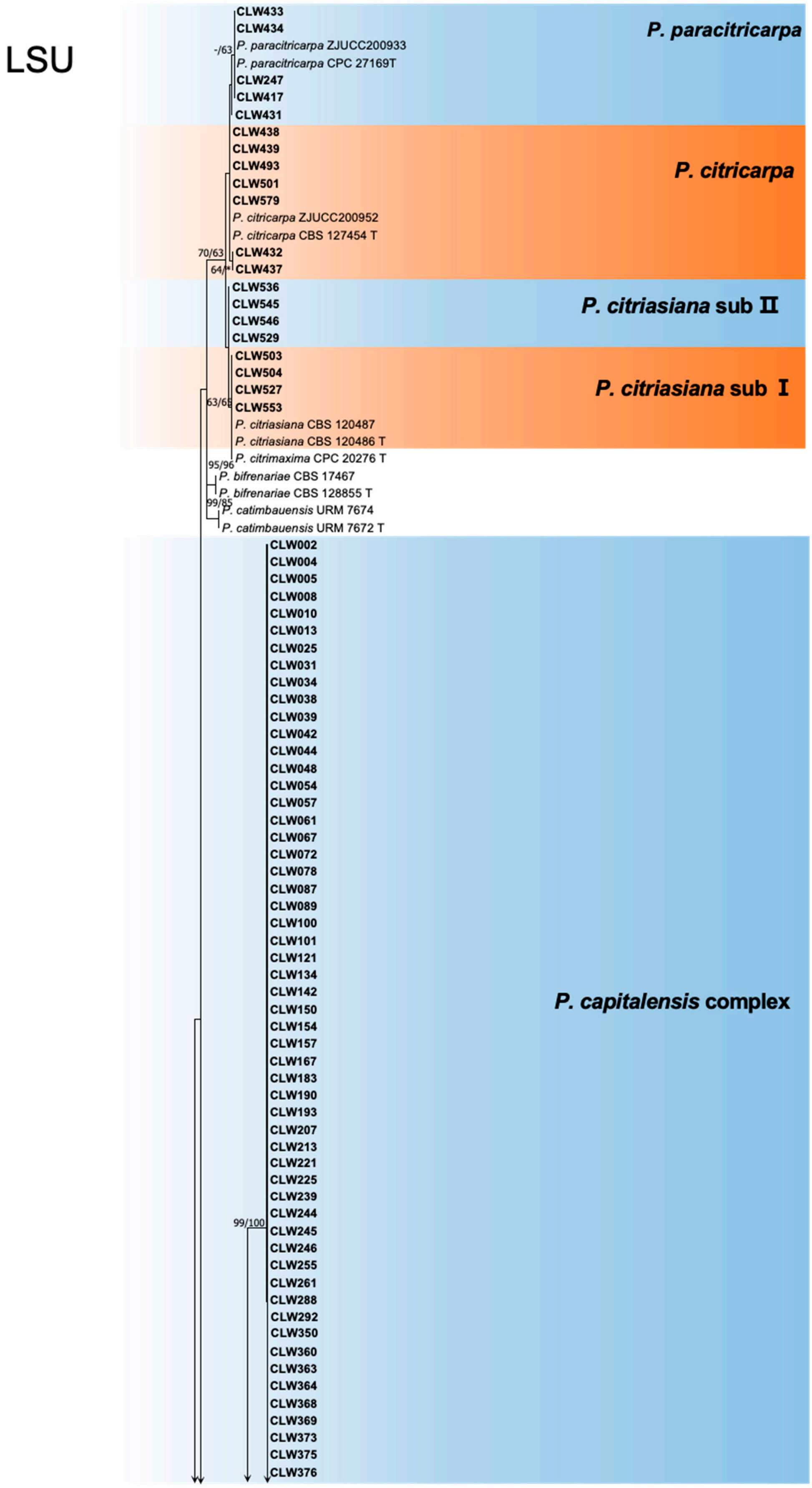


Next page continue.

Continue.

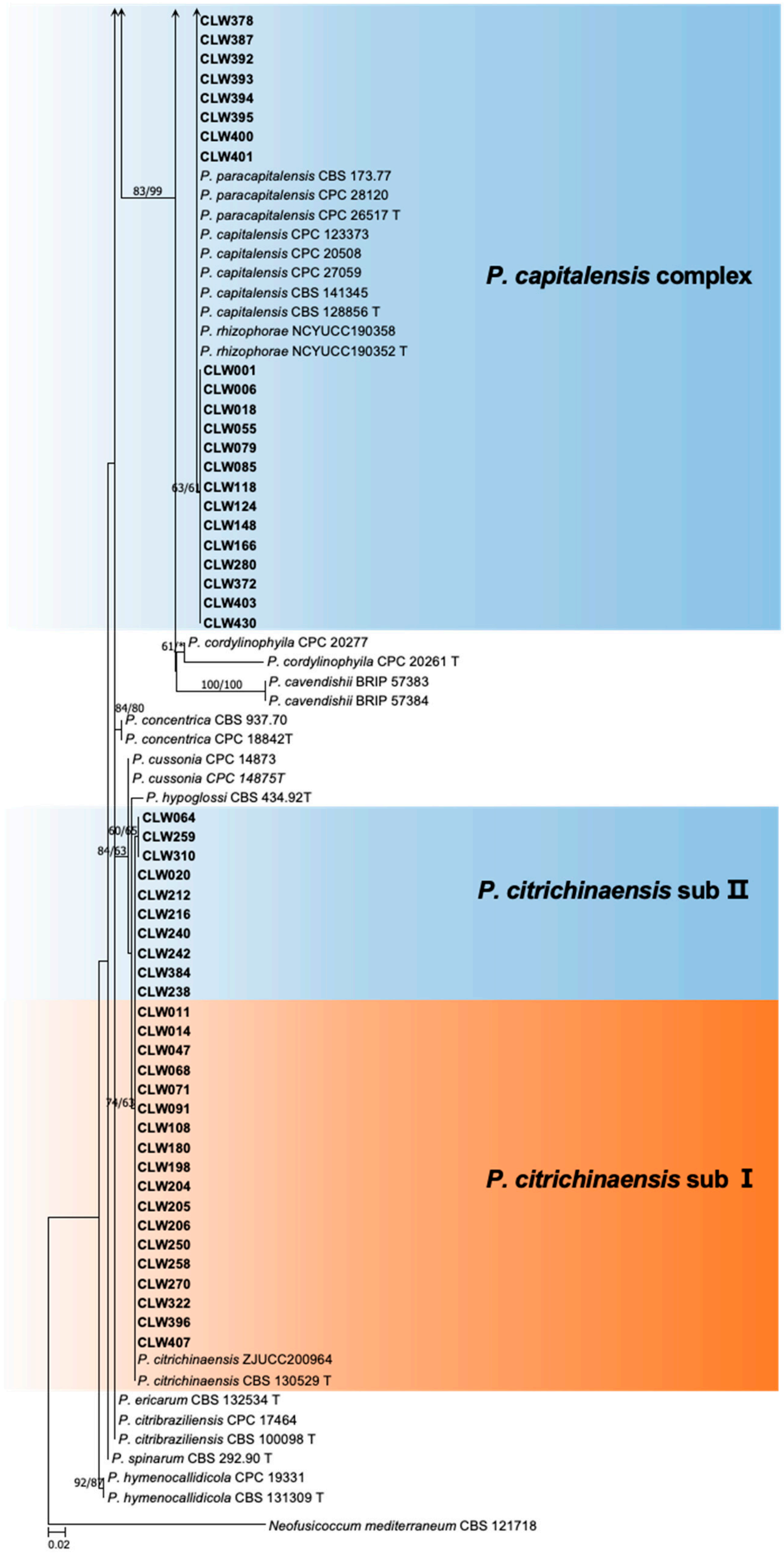


**Figure S6** Maximum likelihood phylogeny of *Phyllostica* isolates related to citrus. 164 isolates of LSU tree.

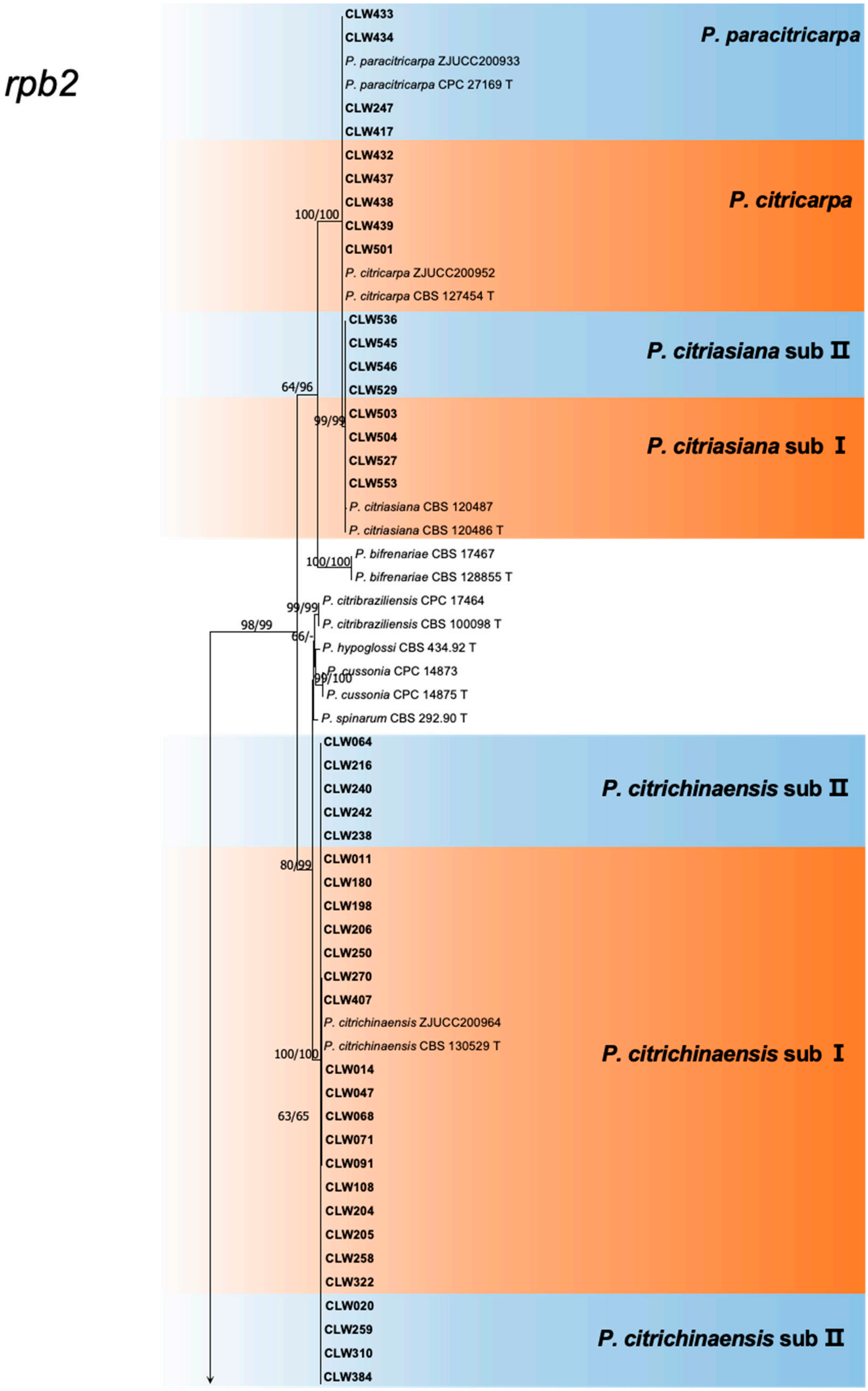


Next page continue.

Continue.

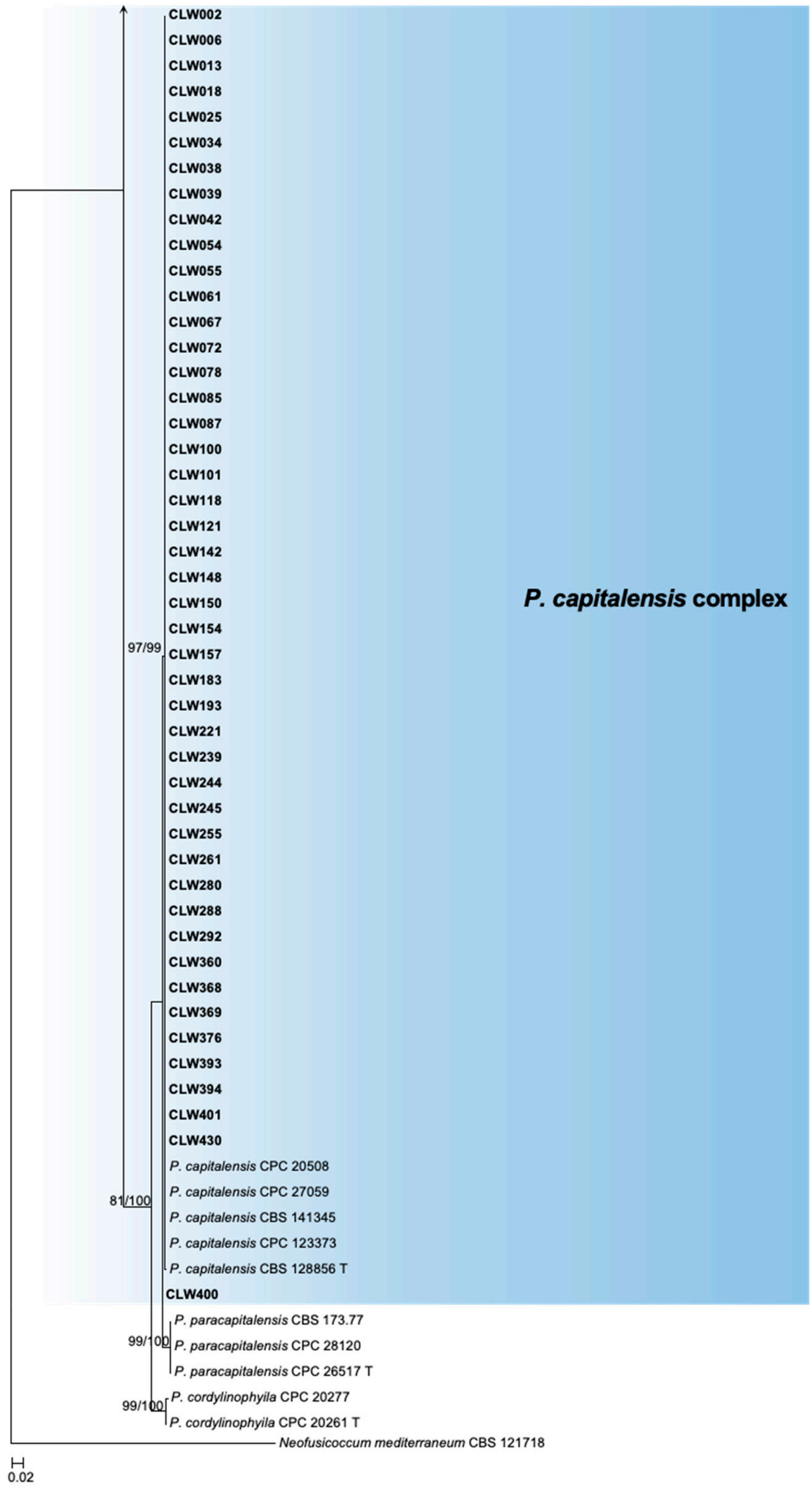


**Figure S7** Maximum likelihood phylogeny of *Phyllostica* isolates related to citrus. 116 isolates of *rpb2* tree. S1-S6, Isolates sequenced in this study are in bold. Bootstrap support values ≥60% for ML and MP are presented above branches as follows: ML/MP, bootstrap values < 60% are marked

with '-', and absent are marked with '*' Ex-type isolates are marked with 'T'. The trees were rooted with *Neofusicoccum mediterraneum* (CBS 121718).

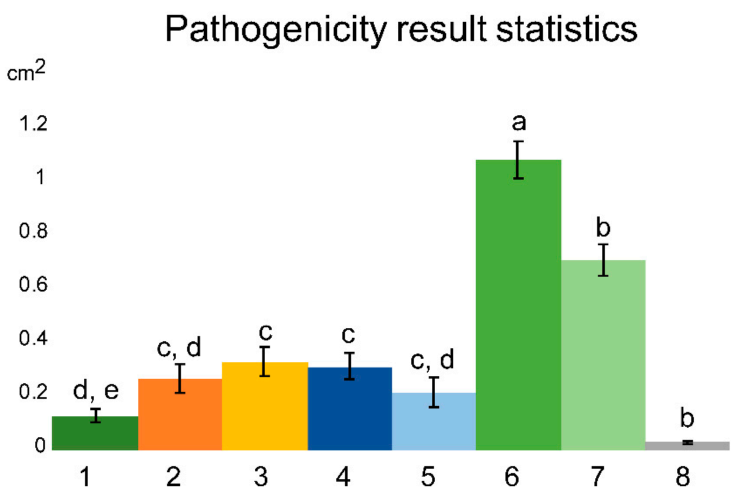


**Figure S8** Column chart indicating the average lesion area produced each isolate of *Phyllosticta* spp. Bar topped with different letters indicate threatment means that are significantly different (P = 0.05). 1 to 7 represent average lesion area caused by isolates of *P. capitalensis, P. citriasiana* sub I & II, *P. citrichinaensis* sub I & II, *P. citricarpa* and *P. paracitricarpa,* 8 represent average lesion area caused by sterile water.
